# Parkin Deficiency Impairs ER-Mitochondria Associations and calcium homeostasis via IP3R-Grp75-VDAC1 Complex

**DOI:** 10.7150/ijbs.121759

**Published:** 2026-01-01

**Authors:** Nai-Jia Xue, Yi Liu, Zhi-Hao Lin, Wen-Hao Huang, Feng Zhang, Ran Zheng, Xiao-Li Si, Lu-Yan Gu, Yi Fan, Jia-Li Pu, Bao-Rong Zhang

**Affiliations:** Department of Neurology, Second Affiliated Hospital, College of Medicine, Zhejiang University, Hangzhou, Zhejiang, 310009, China.

**Keywords:** Parkin, mitochondria-associated ER membrane, calcium, IP3R, ubiquitination

## Abstract

Disruption of mitochondria-associated endoplasmic reticulum membranes (MAMs) and calcium homeostasis has been implicated in the pathogenesis of Parkinson's disease (PD). Parkin, a PD-associated E3 ubiquitin ligase, has been shown to regulate MAM integrity and calcium dynamics. However, the mechanisms of Parkin recruitment and its substrate specificity have not been well understood. This investigation has demonstrated that loss of Parkin enhances ER-mitochondria associations and leads to excessive calcium flux in MAM, resulting in abnormal mitochondrial permeability transition pore (mPTP) opening and decreased cell viability. Further, Parkin physically interacts with IP3R-Grp75-VDAC1 complex at ER-mitochondria contact sites, where it is recruited by IP3R-mediated calcium flux and mitophagy. More importantly, Parkin deficiency leads to the accumulation of IP3R levels, particularly in MAM region. In addition, Parkin fine-tunes the stability of the complex and ubiquitinates IP3R for degradation via the ubiquitin-proteasomal system, ensuring suitable calcium transfer. Taken together, our study reveals a novel role of Parkin in regulating ER-mitochondria contacts, providing insights into PD pathogenesis and potential therapeutic strategies targeting MAMs.

## Introduction

Parkinson's disease (PD) is the second most prevalent neurodegenerative disease characterized by the progressive loss of dopaminergic (DA) neurons in the substantia nigra 1, 2. Most PD cases are sporadic and only 5-10% are familial forms caused by genetic mutations 3. Common mitochondrial, lysosomal, and endosomal dysfunctions observed in both monogenic and sporadic PD 4. These findings underscore that therapies should target the interplay among multiple cellular systems rather than a single molecular factor.

Accumulating evidence shows that the inter-organelle connection between mitochondria and endoplasmic reticulum (ER), known as the mitochondria-associated ER membrane (MAM), plays a crucial role in PD pathogenesis 5, 6. MAMs play critical roles in cellular homeostasis by coordinating trans-organellar calcium signaling, lipid biosynthesis, mitochondrial and ER dynamics, and autophagy. Disruption of MAMs contributes to PD pathogenesis through mitochondrial dysfunction, impaired protein homeostasis, and calcium imbalance, thereby promoting DA neuron degeneration 4, 7. Therefore, maintaining MAM integrity is emerging as a potential therapeutic target for mitigating neurodegeneration in PD.

PD-associated proteins, including LRRK2, PINK1, Parkin, DJ-1, and α-Synuclein, are partly localized to MAMs and may participate in regulating their structure and function, thereby contributing to the pathogenesis of PD 8-11. Among the PD-linked genes, mutations in PRKN, which encodes the E3 ubiquitin ligase Parkin, represent the most common cause of familial PD 12, 13. Parkin cooperates with the mitochondrial kinase PINK1 to mediate ubiquitin-dependent mitophagy 14-16. However, the cellular functions and mechanisms of Parkin transcend mitophagy. Notably, Parkin knockout (KO) mice do not exhibit overt mitochondrial dysfunction, likely due to compensatory mitophagy mechanisms 17-19. Recent evidence indicates that Parkin deficiency heightens neuronal vulnerability to diverse stressors, ultimately causing DA neuron loss and Parkinsonian manifestations 17, 20, 21. Elucidating the mechanisms underlying this increased susceptibility is therefore critical for understanding disease pathogenesis and developing targeted interventions.

Our previous research found that Parkin mutations impair robust pacemaking in induced pluripotent stem cell (iPSC)-derived DA neurons, increasing their susceptibility to stress 22. This vulnerability may result from proteostatic imbalance caused by Parkin deficiency, which in turn disturbs calcium homeostasis 26, 27. About 80% of mitochondrial calcium is transferred from the ER through the IP3R-Grp75-VDAC1 complex at MAMs, supporting mitochondrial metabolism and energy production 23, 24. However, excessive ER-to-mitochondrial calcium flux triggers mitochondrial permeability transition pore (mPTP) opening, leading to apoptosis 24, 25. Given the high metabolic demand of DA neurons, even subtle MAM dysregulation may exacerbate mitochondrial stress and drive their degeneration in PD. Loss of Parkin compromises MAM integrity and disrupts mitochondria-ER communication, thereby amplifying neuronal vulnerability.

## Materials and Methods

### Animals and animal care

Animal care and experiments were conducted in accordance with the National Institutes of Health Guide for the Care and Use of Laboratory Animals and with approval from the Institutional Animal Care and Use Committee of The Second Affiliated Hospital of Zhejiang University (Approval No. 2020-582). Parkin Knockout (KO) (#006582-B6.129S4-Prkntm1Shn/J) C57BL6 mice were from The Jackson Laboratory. Wild-type (Parkin +/+) C57BL6 were obtained from Vital River Laboratory Animal Technology Co. Parkin KO mice were backcrossed with WT mice for more than four generations; homozygous mutant and WT offspring were used for experiments. Mouse genotypes were identified by PCR according to protocol provided by the Jackson Laboratory.

### Cell culture and transfection

Human neuroblastoma M17 control, Parkin KO and Parkin overexpressing (OE) cells were cultured in Opti-MEM medium (Invitrogen) supplemented with 10% fetal bovine serum (FBS) (Invitrogen-Gibco) and 100U/mL penicillin/streptomycin (Invitrogen) at 37°C under 5% CO2. SH-SY5Y control, Parkin KO cells were cultured in DMEM-F12(Invitrogen-Gibco) supplemented with 10% FBS, 1% penicillin-streptomycin. Cells were transfected with Lipofectamine 3000 (Invitrogen) according to manufacturer's instructions. Briefly, plasmid DNA was diluted in Opti-MEM medium and mixed with the P3000 reagent. Lipofectamine 3000 reagent was separately diluted in Opti-MEM medium. The diluted DNA and Lipofectamine solutions were then combined and incubated for 15 minutes to allow formation of DNA-lipid complexes, which were subsequently added to the cells. After incubation for 1-3 days, cells were harvested for further analyses.

### siRNA-mediated knockdown

For siRNA transfection, we used Lipofectamine RNAiMAX (Invitrogen). PINK1 knockdown was achieved by using siRNAs against PINK1 obtained from Santa Cruz (sc-44598). Briefly, siRNA and Lipofectamine RNAiMAX were each diluted in Opti-MEM, then combined and incubated for 20 min at room temperature to form complexes, which were added to the cells. Cells were analyzed 3 days after transfection.

### Generation of Parkin KO and Parkin OE cells

Lentivirus (LV) containing Parkin guide RNA sequence (GTGTGACAAGACTCAATGAT) and backbone plasmids, which were purchased from Vigene Biosciences (Shandong, China) were transduced. LV containing pCDH-CMV-MCS-EF1-CoGFP-T2A-Puro-PRKN, which were purchased from GENERAL BIOL (Anhui, China) were transduced. Twenty-four hours after infection, transfected cells were selected by 0.5 μg/ml puromycin for 3 days, and then single cells were transferred onto 96-well plates with one colony in each well.

### Antibodies and chemicals

Parkin (4211S, 1:1000 for WB) and SigmaR1 (D4J2E, 1:1000 for WB) antibodies were purchased from Cell Signaling Technology. IP3R (A7905, 1:1000 for WB and BN-PAGE) and VDAC1 (A19707, 1:1000 for WB, 1:200 for PLA) and COX IV (A11631, 1:1000 for WB) and PINK1 (A24745, 1:1000 for WB) were purchased from ABclonal. Tyrosine Hydroxylase (ab76442, 1:500 for immunostaining) antibodies were purchased from Abcam. Parkin (sc-32282, 1μg for IP, 1:300 for BN-PAGE), IP3R (sc-377518, 1:100 for PLA, 1μg for IP) and Grp75 (sc-133137, 1:1000 for WB and BN-PAGE, 1:100 for PLA) antibodies were purchased from Santa Cruz Biotechnology. Parkin (ET1702-60, 1:200 for PLA) antibodies were purchased from HUABIO. HA (B1021, 1:5000 for WB) antibodies were purchased from Biodragon. VDAC1 (55259-1-AP, 1:1000 for BN-PAGE) and GAPDH (10494-1-AP, 1:5000 for WB) antibodies were purchased from Proteintech. Mouse IgG (A7028) and Rabbit IgG (A7016) were purchased from Beyotime. Alexa Fluor 488-conjugated anti-rabbit (A-11008, 1:500), Alexa Fluor 594-conjugated anti-mouse (A-11032, 1:500) and Alexa Fluor 488-conjugate anti-chicken (A-11039, 1:500) were purchased from Invitrogen. N, N-Dimethylacrylamide (DMA, #27413-5) and CHX (C7698) were purchased from Sigma-Aldrich. Carbonyl cyanide m-chlorophenyl hydrazone (CCCP, S6494), MG-132 (S2619), Thapsigargin (TG, S7895) and 2-Aminoethyl Diphenylborinate (2-APB, S6657) were obtained from Selleck. Lipofectamine 3000 (L3000015) was purchased from Invitrogen. Human Parkin WT, C431S, T240R and R275W mutant cloned into pCDH-CMV-MCS-EF1-CoGFP-T2A-Puro vector were obtained from YouBio (China).

### Measurement of basal mitochondrial calcium levels and mPTP

Control and Parkin KO SH-SY5Y and M17 cells were seeded in 6 well plate. For measurement of basal mitochondrial calcium level, cells were stained with 4 µM Rhod2/AM (Invitrogen) in cell media for 30 min at 37 °C in a humidified atmosphere composed of 5% CO_2_. The mitochondrial permeability transition pore was measured by the Mitochondrial Permeability Transition Pore Assay Kit (Beyotime, C2009S). Calcein-AM fluorescence is retained in both the mitochondria and cytosol. CoCl₂ selectively quenches cytosolic Calcein-AM fluorescence, while mitochondrial fluorescence remains intact. Upon mitochondrial permeability transition pore (mPTP) opening, cobalt ions enter the mitochondria, leading to quenching of the mitochondrial Calcein-AM fluorescence as well. Cells were incubated with 1 ml binding buffer containing 1 µl Calcein-AM and 10 µl CoCl₂ for 30 min at 37 °C in a humidified atmosphere with 5% CO₂. The samples were measured by flow cytometry (BECKMAN, CytoFlex LX).

### Measurement of dynamic calcium influx and efflux in subcellular compartments

Control and Parkin KO SH-SY5Y and M17 cells were cultured on 10 mm poly-D-lysine (ST508, Beyotime) coated 35mm confocal dish (J40141, JingAn Biological). For measurement of mitochondrial calcium level, cells were stained with 4 µM Rhod2/AM (Invitrogen) in cell media for 30 min at 37 °C in a humidified atmosphere composed of 5% CO2. For measurement of ER calcium level, cells were transient transfected with CMV-ER-LAR-GECO1 (Plasmid #61244, Addgene). We monitored the live cells using LSM900 laser scanning confocal microscopy (Carl Zeiss, Germany). The culture medium was replaced by warm 20mM HEPES buffered HBSS (HHBSS) buffer. After 1 minute of baseline recording, a single pulse of TG was delivered to liberate calcium stores for 3 min and then washed out. Peak amplitudes of Ca^2+^ responses to TG were normalized to the basal fluorescence (F0) before stimulation. The area-under-the-curve (AUC) of the bar graph was calculated by multiplying the changes in fluorescence over the basal (ΔF/F0) by the time. Calcium transients were continuously recorded and analyzed by Time Series Analyzer on Fiji (NIH, java6).

### Cell viability assay

Cell viability assay was performed with a Cell Counting Kit-8 (CCK-8, Beyotime, C0038) according to the manufacturer's instructions. Briefly, cells were seeded in 96-well plates and treated with TG, the CCK-8 solutions were added and incubated for 4 h. Absorption at 450 nm was subsequently measured on a microplate reader (BIOTEK, ELX-808). Each experiment was repeated three times individually. The results were expressed as a percentage of control, which was set at 100 %.

### Apoptosis determination

Cells were first collected by removing the culture medium and then trypsinized to digest the cells. After digestion, the supernatant was collected, and the cells were washed three times with cold PBS. They were then resuspended in 200 μl of 1× binding buffer containing 5 µl Annexin V-FITC and 10 µl propidium iodide (PI) (Beyotime, C1062L). After incubation for 20 min at 25 °C in the dark, an additional 200 µl of 1× binding buffer was added to each tube, and the samples were analyzed by flow cytometry (BECKMAN, CytoFlex LX).

### Proximity ligation *in situ* assay

The Duolink^®^ *in situ* ligation assay (PLA) enables evaluation of the two proteins and their potential interaction (< 40nm) as an individual dot under microscopy. PLA was performed using Duolink^®^ reagents (Sigma-Aldrich) following the manufacturer's instructions. Briefly, samples (cells and tissues) fixation, permeabilization and blocking were performed, were then incubated overnight with paired primary antibodies at 4 °C. After buffer washed, we applied combinations of the corresponding *in situ* PLA oligonucleotide probes (anti-rabbit PLUS and anti-mouse MINUS) for 1 h at 37 °C. After ligation, the signal was amplified by rolling circle amplification (RCA) under the action of polymerase to form dot-like PLA signals which can be captured under fluorescence microscope. The samples were incubated with polymerase for 100 min. Preparations were mounted in Duolink^®^ mounting medium containing DAPI.

For PLA performed on tissue sections, mouse brain samples containing substantia nigra (SN) were fixed in 4% PFA embedded. 10μm thick tissue sections of SN were used for PLA staining. Finally, samples (cells and tissues) were scanned by LSM900 laser scanning confocal microscopy (Carl Zeiss, Germany).

### Electron microscopy

Electron microscopic (EM) analysis of M17 cells and substantia nigra was performed as previously reported 26. Cells were fixed with 2.5% glutaraldehyde in PBS (pH 7.2) overnight at 4 ℃. Subsequently, the cells were refixed in 1% osmium tetroxide for 1 h, stained with 2% uranyl acetate for 30 min, and dehydrated in a series of graded ethanol. Next, the samples were embedded in 100% acetone for 2 h, sliced into 90 nm ultrathin sections, and stained with 4% uranyl acetate and 1% lead citrate. The EM (Thermo Scientific Talos 120kV, American) was used to evaluate the ultrastructure of cells. The images were analyzed manually for the ER-Mitochondrial using ImageJ software (NIH, java8). The scale of image was set using Set Scale tool on ImageJ. Followed by measuring the length of ER or perimeter of mitochondria using freehand line tool. MAM length is defined as the length of ER in regions where the ER-mitochondria distance is less than 30 nm. MAM distance refers to the average distance between the ER and mitochondria within the MAM region (within contact distances of 30 nm). The MAM coverage was determined by taking the ratio of the length of MAM length to the perimeter of mitochondria within a single cell.

### Total RNA isolation and real-time PCR

Total RNA was extracted from M17 cells and mouse brain with the RNA/DNA Co-isolation Kit (R0017S, Beyotime, China) according to the manufacturer's instructions. TB Green Premix Ex Taq II (Takara, RR420A) for qPCR was used with the StepOnePlus Real-Time PCR System (Applied Biosystems). The following primer sequences were used for RT-qPCR.

### Western blotting (WB)

Immunoblotting was performed according to standard procedures. Briefly, the tissues and cells were extracted by ice-cold lysis RIPA buffer supplemented with phosphatase and protease inhibitors for 30 min. After centrifugation at 12000×g for 15min at 4 °C, cell debris was discarded, and supernatant was kept on ice. Protein concentration was measured by BCA protein assay kit (P0012, Beyotime). Equal protein concentration in each sample was prepared and 5X SDS-PAGE Sample Loading Buffer was added and boiled at 100 °C for 5 min. Protein samples with equal amounts of protein were separated by sodium dodecyl sulfate-polyacrylamide gel electrophoresis (SDS-PAGE) and transfered onto polyvinylidene difluoride membranes (PVDF) under constant current of 250 mV for 120 min condition. Membranes were incubated with primary and HRP-conjugated secondary antibodies and visualized using a chemiluminescent reagent. The gray density of the signals on the brands was quantified using the ImageJ software (NIH, java8).

### Co-localization upon volume-rendered 3D reconstruction

Mitochondria and ER were marked by Mito-Tracker Deep Red FM (C1032, Beyotime) and ER-Tracker Green (C1042S, Beyotime) following a published protocol. Briefly, Control and Parkin KO SH-SY5Y and M17 cells were cultured on 10 mm poly-D-lysine (ST508, Beyotime) coated 35mm confocal dish (J40141, JingAn Biological). The cells were washed with warm HBSS buffer and then incubated in 20 nm Mito-Tracker Deep Red FM and 1:1000 ER-Tracker and Green dissolved in HBSS for 30min. Cells were washed HBSS twice and then scanned by LSM900 laser confocal microscope (Carl Zeiss, Germany). The percentage of mitochondria that colocalize with ER was measured with Pearson's coefficient of colocalization (JACoP, imageJ), following 3D volume rendered reconstruction of z-axis images separated by 0.16 μm on Zen software (Carl Zeiss, Germany).

### Subcellular fractionation

Brain tissues and cells fractionations were performed following a published protocol 27. Briefly, cells were washed and prepared by centrifugation at 600 g for 5 min, resuspended in IBcells-1 buffer (225 mM mannitol, 75mM sucrose, 0.1 mM EGTA and 30 mM Tris-HCl pH7.4) and gently repeated strokes in Dounce homogenization (Sigma-Aldrich). Homogenization was monitored by Trypan Blue staining, and strokes were stopped when approximately 80% of cells were disrupted. The homogenate was centrifuged at 600 g for 5 minutes for several times to remove nuclei and unbroken cells. Then the supernatant was centrifuged at 7000g for 10 minutes to obtain crude mitochondria (CM, pellet).

The resultant supernatant was centrifuged at 20000g for 30 min at 4℃. The pellet consists of lysosomal and plasma membrane fractions. Then the supernatant was centrifugated at 100 000 g for 90 min (MLS50 rotor, Beckman) at 4℃ to isolate ER (pellet). The CM fraction, resuspended in MRB (mitochondria resuspending buffer, 250mM mannitol, 5mM HEPES pH 7.4 and 0.5 mM EGTA), was subjected to Percoll gradient centrifugation (Percoll medium: 225mM mannitol, 25mM HEPES pH 7.4, 1mM EGTA and 30% vol/vol Percoll) in a 5-ml Ultra-Clear tube (344057, Beckmam). After centrifugation at 95000 g for 30 min (MLS50 rotor), a dense band at the bottom of the tube containing purified mitochondria was recovered, washed by centrifugation at 6300g for 10 min to remove the Percoll and finally resuspended in MRB. The MAMs were removed from the Percoll gradient as a diffuse white band located above the mitochondria, were diluted in MRB and centrifuged at 6300 g for 10 min to remove mitochondrial contamination; then the supernatant was further centrifuged at 100000 g for 90 min (MLS50 rotor, Beckman) to pellet the MAMs fraction.

### Co-immunoprecipitation (Co-IP)

Co-immunoprecipitation (Co-IP) was performed using the Dynabeads Protein G Kit (Invitrogen), following the manufacturer's protocol. Cells and mouse brain were solubilized in NP-40 Lysis Buffer (P0013F, Beyotime) with protease inhibitors. For immunoprecipitation, 1μg primary antibodies or control IgG were incubated with 50μl Dynabeads at room temperature for 30 min. The supernatant was then incubated with indicated antibody coated Dynabeads at 4 ℃ overnight. Beads were washed four times with wash buffer. After washing, proteins were eluted, boiled in SDS-PAGE sample buffer, and analyzed by immunoblotting.

### Ubiquitination assays

IP3R was isolated by immunoprecipitation under denaturing conditions to disrupt protein complexes 28. Whole cell lysates were lysed in SDS lysis buffer (2% SDS, 150 mM NaCl, 10 mM Tris-HCl (pH = 8.0)) with protease inhibitor mixture, a phosphatase inhibitor, and 10 μM N-ethylmaleimide (NEM, HY-D0843, MCE). Lysates were immediately heated for 10 min at 100° and then were diluted ten times with dilution buffer (10 mM Tris-HCl, pH 8.0, 150 mM NaCl, 2 mM EDTA, 1% Triton). The following steps were the same as the Co-IP assay described above.

### Mass spectrometry (MS) analysis

Parkin protein was immunoprecipitated as described above for Co-IP. MS analysis was performed by Cosmos Wisdom (Hangzhou, China). Briefly, Gel samples were stained with Commassie blue, and bands of interest were excised into approximately 1mm³ slices. The slices underwent destaining, drying, reduction, alkylation, and dehydration processes. Proteins were digested with trypsin and prepared for LC-MS/MS analysis on a Q Exactive mass spectrometer (Thermo Fisher Scientific). The peptide samples were separated and analyzed under specific conditions, with the data acquired using a data-dependent top10 method. Proteome Discoverer 2.2 and Mascot 2.6 engines were used for protein identification against the UniProt database using the following parameters: maximum number of missed cleavages, 2; precursor tolerance, 20 ppm; dynamic modification, oxidation (M) and N-terminal protein acetylation, deamidated (NQ); static modification, carbamidomethyl (C); and false discovery rate, < 0.01.

M17-Parkin cells were treated with 10 µM MG-132 for 6 h, and IP3R was enriched via ubiquitination assays. Gel bands corresponding to IP3R were excised, destained with 50% acetonitrile/50 mM NH₄HCO₃, dehydrated, reduced with 10 mM TCEP, and alkylated with 40 mM CAA at 60 °C for 30 min. After dehydration, gel pieces were digested with 2 µg trypsin in 50 mM NH₄HCO₃ at 37 °C overnight. Peptides were extracted with 50% acetonitrile/0.1% formic acid and separated on an EASY-nLC 1200 at 400 nl/min. Eluted peptides were analyzed on a Q Exactive HF-X MS, with MS1 at 60,000 and MS/MS at 15000 resolution. Data were processed using Proteome Discoverer 2.4 against the human SwissProt database, with Trypsin/P cleavage, carbamidomethylation as fixed, and oxidation/acetylation as variable modifications. Ubiquitination proteomics analysis was performed by Cosmos Wisdom (Hangzhou, China).

### Blue native (BN) and SDS-PAGE 2D separation

Blue native and 2D PAGE were performed according to the user guide of Novex® native gel electrophoresis system (Life Technologies). Briefly, crude mitochondria were lysed in 1%NP40 buffer to obtain the native lysates. The lysates were centrifuged at 10000 for 10 min at 4℃ to remove unsolubilized debris. Protein concentration was determined by BCA Protein Assay Kit (P0012, Beyotime). 1st dimension Blue-Native PAGE was performed according to manufacturers' instructions (Invitrogen). 30 μg of proteins combined with 0.25% G-250 was loaded onto a 3-12% Bis-Tris gel (Invitrogen). Native PAGE was performed at 120 V for 30 min until the dye front reached one-third of the gel length, after which the Dark Blue Cathode Buffer was replaced with Light Blue Cathode Buffer. The gel was then run at 120 V for an additional 2 h to achieve protein separation. After 1st dimension electrophoresis, proteins were transferred to a PVDF membrane following the standard WB transfer procedure. The membrane was then incubated in 20 ml of 8% acetic acid for 10 min to fix the proteins.

For two-dimensional electrophoresis, at the end of the first dimension, the gel strips were excised and incubated for 15 minutes at room temperature in 1×NuPAGE LDS sample buffer with 1×NuPAGE reducing agent (Invitrogen) to reduce disulfide bonds. Then, cysteines alkylation was done with DMA for 15min at RT. Finally, the reaction was quenched for 15min at RT with 20% ethanol in 1X LDS and 0.1X Reducing agent. The equilibrated gel strip was then immediately applied to the second dimension on a 2D 4-16% Bis-Tris SDS gel (Invitrogen). The gel was run at 120 V for approximately 2 hours to separate denatured proteins by size, after which the proteins were transferred to a PVDF membrane.

## Results

### Loss of Parkin increases ER-mitochondria contact sites

**Figure 1:**
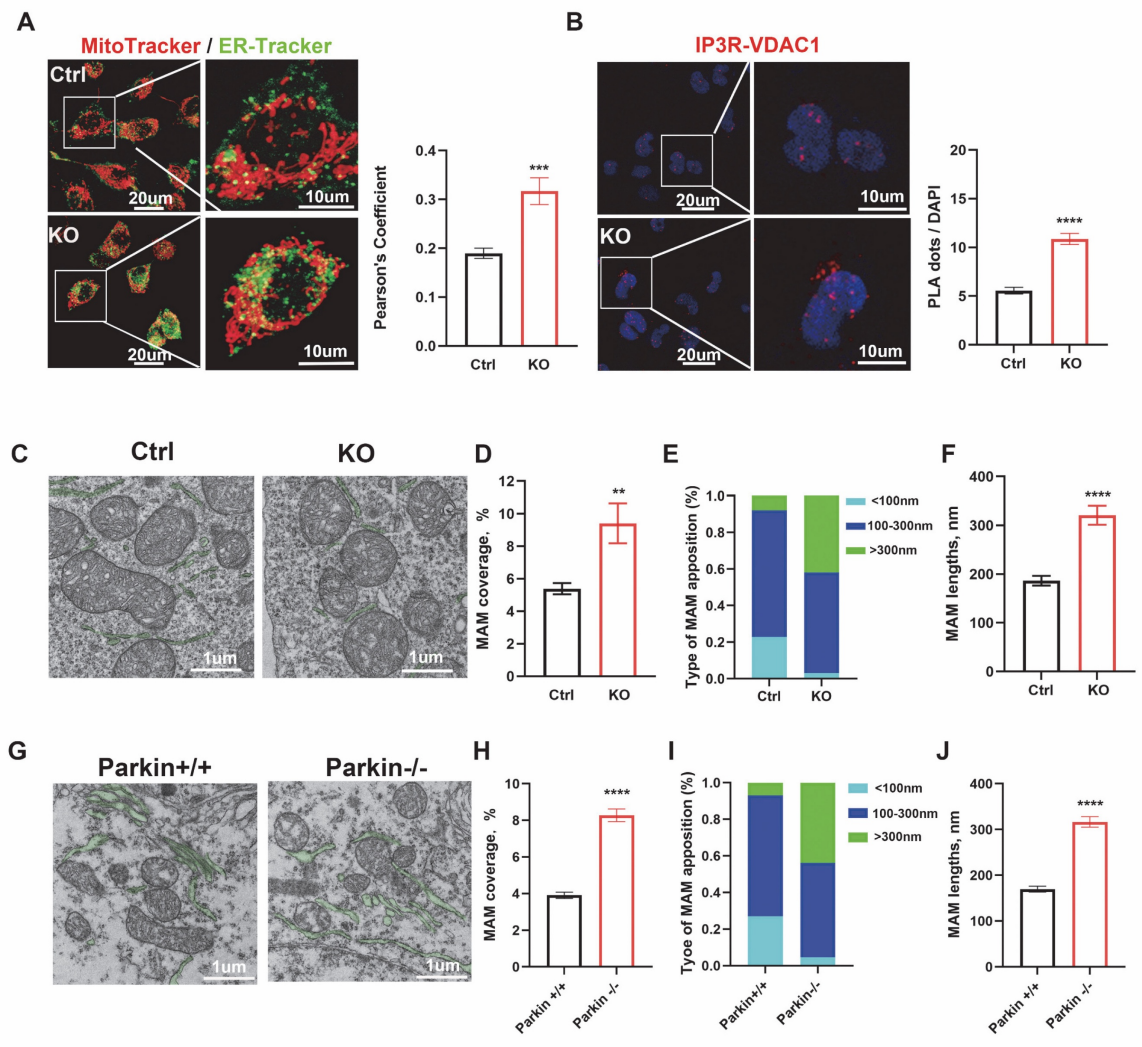
** Loss of parkin increases endoplasmic reticulum (ER)-mitochondria contact sites.** (A) Confocal microscopy was used for live cell imaging of both control cells (upper) and Parkin KO M17 cells (bottom). ER was stained with an ER-Tracker (green) and mitochondria were stained with MitoTracker (red) (Scale bar, 20 μm). Quantification of ER-mitochondria association was performed using ImageJ (bar graphs on the right). (B) Confocal microscopy images of proximity ligation assays (PLA) of ER-mitochondria association. PLA signals (red dots) were determined by interaction between IP3R and VDAC1 in control cells (upper) and Parkin KO M17 cells (bottom) (Scale bar, 20 μm). PLA red fluorescent dots were present as the number of positive interactions per nucleus (bar graphs on the right). (C) Electron micrographs of mitochondria connected to the ER (pseudocolored green) in control and Parkin KO M17 cells (Scale bar, 1 μm). (D-F) Ultrastructural analysis of ER-mitochondria association. Quantitative analysis of the percentage of the mitochondria-associated endoplasmic reticulum membrane (MAM) to mitochondria (D), type of MAMs apposition (F) and average length (I) of ER-mitochondria association in control cells (n = 7) and Parkin KO M17 cells (n = 8). (G) Electron micrographs of mitochondria connected to the ER (pseudocolored green) in neurons from wild-type (WT) and Parkin KO mouse brains. (H-J) Ultrastructural analysis of ER-mitochondria association. Quantitative analysis of the percentage of the MAM to mitochondria (H), type of MAM apposition (I), and average length (J) of ER-mitochondria association (n = 5 mice per group, with 4 neurons per mouse). Data are expressed as means ± SEM based on three independent experiments. Data were analyzed using a two-tailed unpaired Student's t-test. *P < 0.05; **P<0.01; ***P < 0.001.

To study the role and mechanism of loss-of-function of Parkin in ER-mitochondria contacts, Parkin KO monoclonal lines of M17 and SH-Y5Y cells were generated using the CRISPR/Cas9 gene-editing (Fig. S1A-B). Firstly, the ER-mitochondria associations were scrutinized using various methods. Mitochondrial and ER fluorescent markers (Mito-Tracker Deep Red FM and ER-Tracker Green) were introduced into live cells to visualize ER and mitochondria respectively. ER-mitochondria contacts increase markedly in Parkin KO cells (Fig. 1A and Fig. S1C). *In situ* proximity ligation assay (PLA) is a sensitive method to detect protein colocalization < 40 nm away from each other, which indicates protein interaction. Thus, ER-mitochondria contacts were visualized by IP3R/VDAC1 PLA signals 29. Compared with control cells, IP3R/VDAC1 PLA signals increased significantly in Parkin KO M17 and SH-SY5Y cells (Fig. 1B and Fig. S1D). Meanwhile, electron microscopy (EM) directly measured the ER-mitochondria morphology (Fig. 1C and Fig. S1F), confirming that the association was markedly elongated in Parkin KO cells. In M17 cells, MAM coverage increased from 5.38 ± 0.34% in controls to 9.18 ± 1.09% in Parkin KO cells (Fig. 1D), accompanied by a higher proportion of long (> 300 nm) contacts (Fig. 1E) and a longer average ER-mitochondria association (321.0 ± 19.39 nm vs. 186.6 ± 10.01 nm) (Fig. 1F). No significant change was observed in the inter-organelle distance (Fig. S1E). Similar results were obtained in SH-SY5Y cells, where MAM coverage increased from 4.87 ± 0.35% to 11.28 ± 1.39% (Fig. S1G), with more long contacts (Fig. S1H) and a longer average ER-mitochondria association (431.9 ± 31.0 nm vs. 198.0 ± 13.02 nm) (Fig. S1I), while the ER-mitochondria distance remained unchanged (Fig. S1J).

These concepts were also tested in neurons of the substantia nigra in mouse brain. We assessed the MAMs coverage and the average length of MAMs in wild-type (WT) and globally Parkin-KO mice using EM (Fig. 1G). The MAMs coverage in Parkin KO neurons was significantly higher, measuring 8.20 ± 0.34%, compared to 3.91 ± 0.17% in WT neurons (Fig. 1H). Additionally, the longer ER-mitochondria association (>300 nm) exhibited a significant increase (Fig. 1I), with the average length of MAMs in Parkin KO dopaminergic neurons measuring 316.60 ± 11.52 nm, compared to 169.80 ± 6.45 nm in wild-type dopaminergic neurons (Fig. 1J). No changes in the distance between the ER and mitochondria were noted (Fig. S1K). Thus, silencing Parkin increases ER-mitochondria associations *in vitro* and *in vivo*.

### Parkin knockdown induces mPTP opening via MAMs-mediated Ca^2+^ influx

**Figure 2:**
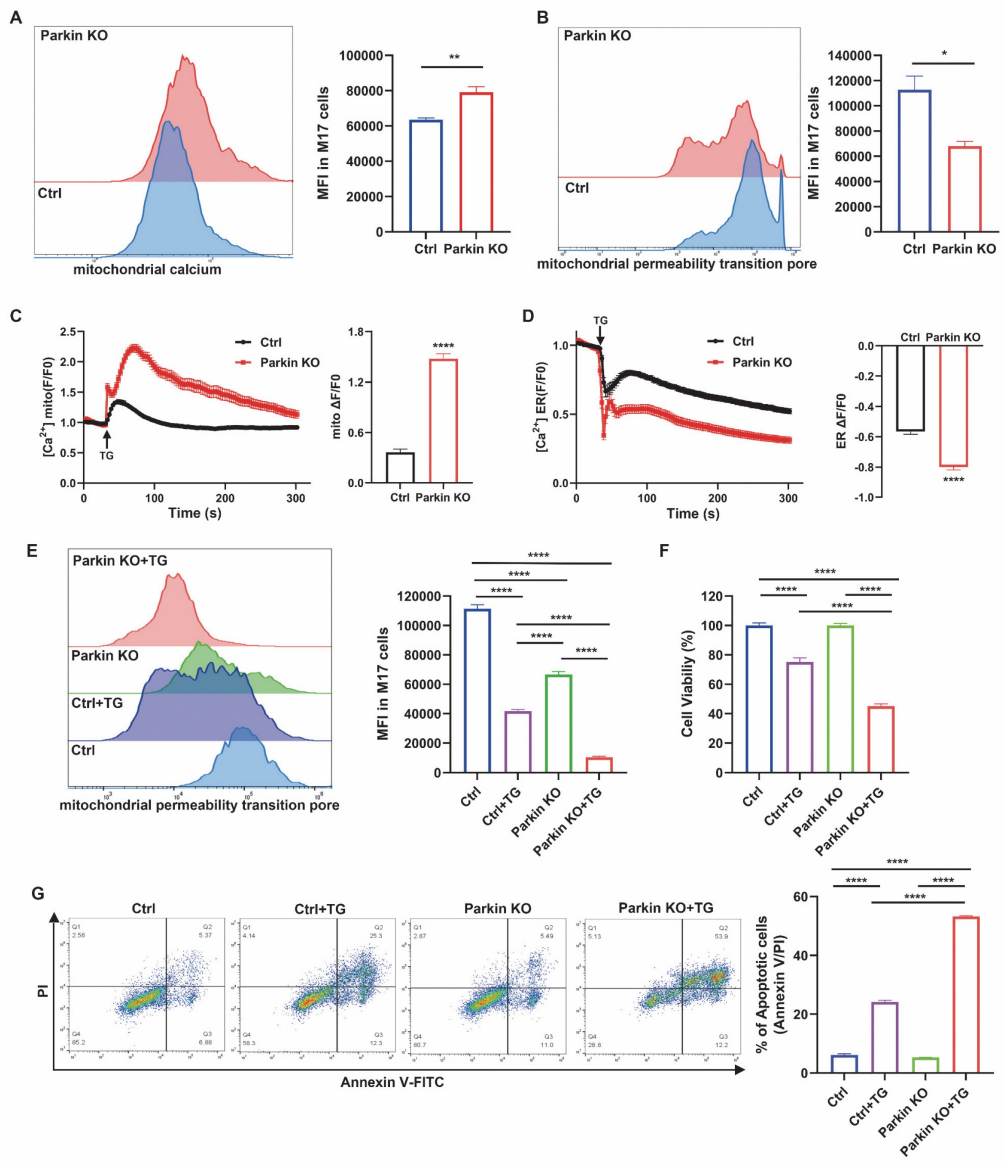
** Parkin knockdown induces mPTP opening via MAM-mediated Ca^2+^ influx.** (A) Mitochondrial calcium levels were evaluated using Rhod-2 staining and quantified by mean fluorescence intensity (MFI) through flow cytometry comparisons. (B) The mPTP functionality was gauged with calcein-AM staining in conjunction with CoCl2. (C and D) Measurement of calcium modulation in mitochondria (C) and the ER (D) in control cells (black) and Parkin KO M17 cells (red) was conducted using confocal microscopy. Thapsigargin (TG) was used to initiate calcium release. The right bar graphs indicate the quantification of maximal mitochondrial (C) or ER (D) calcium peak fluorescence during TG treatment (n = 68-83 cells). (E) Treatment with TG (2.5μm, 24 hours) in both genotypes was followed by an analysis of mPTP functionality using calcein-AM fluorescence. (F) CCK-8 assay was used to examine cell viability across four groups after treatment with TG (2.5μm, 24 hours). (G) Apoptosis was analyzed by flow cytometry using Annexin V/PI (propidium iodide) staining, following treatment with TG (2.5 μM, 24 hours). Data are expressed as means ± SEM based on three independent experiments. Data were analyzed using two-tailed unpaired Student's t-test (A-D) and one-way analysis of variance (ANOVA) with Tukey's multiple comparisons test (E-F). *P < 0.05; **P<0.01; ***P < 0.001.

At baseline, mitochondrial calcium levels assessed by Rhod-2 AM staining showed a moderate increase in Parkin KO M17 and SH-SY5Y cells (Fig. 2A and Fig. S2A). Parkin deficiency also caused a slight elevation in mPTP opening, as indicated by calcein-AM fluorescence quenching after CoCl₂ treatment (Fig. 2B and Fig. S2B). To further examine calcium handling, cells were treated with TG, which blocks the ER Ca²⁺-ATPase to induce ER calcium release 30. Upon TG stimulation, Parkin KO M17 and SH-SY5Y cells exhibited markedly enhanced mitochondrial calcium uptake compared with controls (Fig. 2C and Fig. S2C). Consistently, ER calcium release monitored by CMV-ER-LAR-GECO1 was significantly increased in both Parkin KO cell lines (Fig. 2D and Fig. S2D). The enhanced mitochondrial calcium influx was accompanied by pronounced mPTP opening (Fig. 2E and Fig. S2E), reduced cell viability, and increased apoptosis (Fig. 2F-G and Fig. S2F-G). Collectively, these findings demonstrate that Parkin loss disrupts MAM-mediated calcium homeostasis, rendering cells more susceptible to calcium-induced stress through aberrant mPTP opening.

### Parkin resides in the MAMs and interacts with the IP3R-Grp75-VDAC1 complex

**Figure 3:**
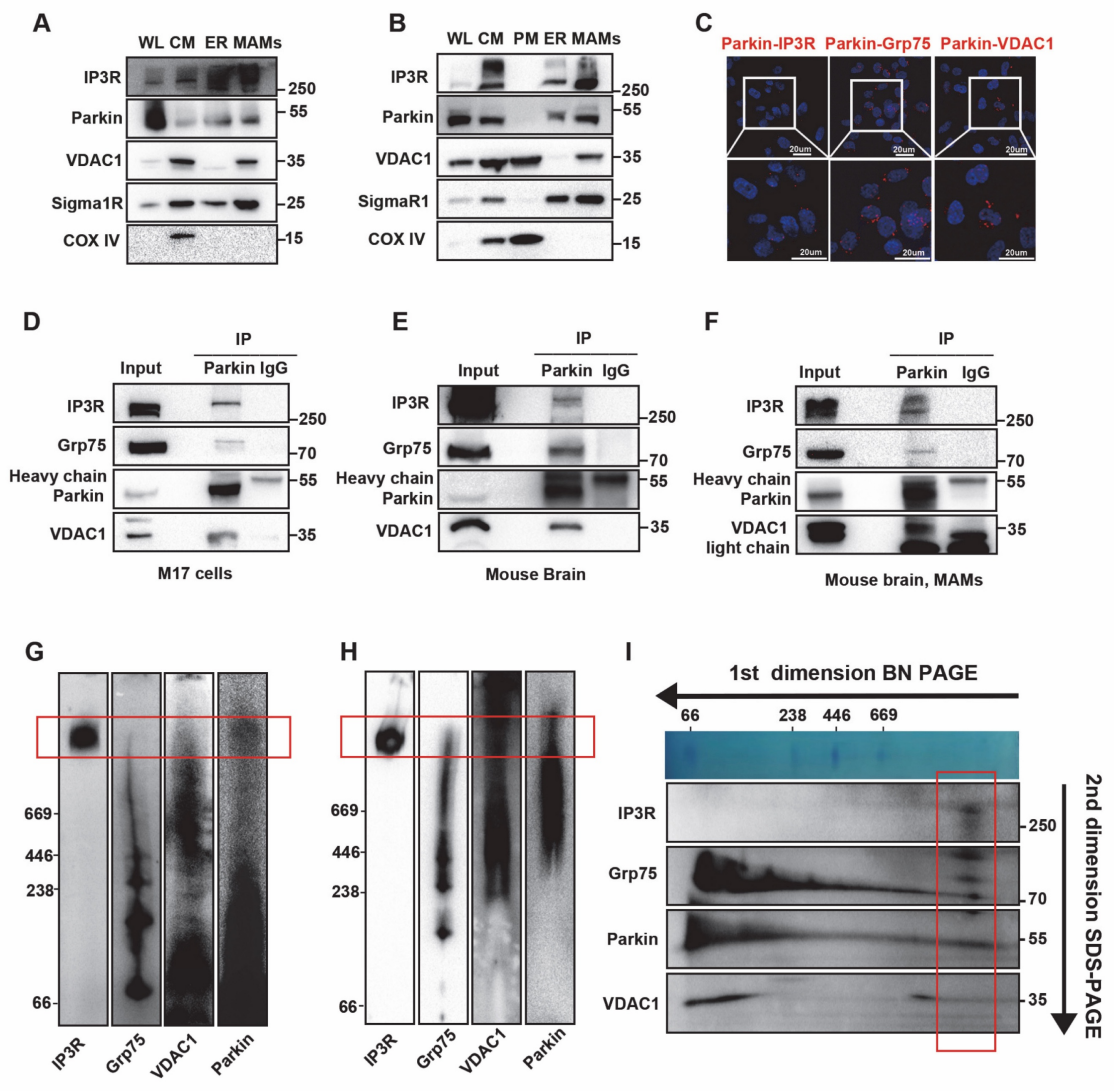
** Parkin resides in the MAM and interacts with the IP3R-Grp75-VDAC1 complex.** (A-B) Subcellular fractionation and immunoblot characterization of parkin in the MAM fraction in wild-type C57BL6 mouse brain (A) and parkin-overexpressing M17 cells (B). Cells were fractionated into whole lysates (WL), crude mitochondria (CM), pure mitochondria (PM), ER, and MAM. Three independent experiments were conducted. (C) *In situ* close association between Parkin/IP3R (left), Parkin/Grp75 (middle), and Parkin/VDAC1 (right) were determined by proximity ligation assay (PLA) in normal M17 cells (Scale bar, 20 μm). (D-F) Immunoblot analysis of IP3R, Grp75, and VDAC1 in the parkin immunoprecipitates of total lysates from parkin-overexpressing M17 cells (D) and ventral midbrain of mouse brain (E). MAM fractions were prepared from the ventral midbrain of the mouse brain (F). Three independent experiments were conducted. (G-H) Blue native-polyacrylamide gel electrophoresis (BN-PAGE) and immunoblot analysis of CM fractions prepared from parkin-overexpressing M17 cells (G) and ventral midbrain of mouse brain (H) showed a large protein complex. The red box in G-H highlights the macrocomplex that contains all four components. Three independent experiments were conducted. (I) Two-dimensional separation and immunoblot analysis of CM fraction from parkin-overexpressing M17 cells showed a large protein complex. The red box in I highlights the macrocomplex that contains all four components. Three independent experiments were conducted.

Next, we assessed whether Parkin is an endogenous MAMs protein to further explore the underlying mechanism by which Parkin regulates the ER-mitochondria association. Mitochondria, ER, and MAMs fractions were sequentially purified via Percoll-based subcellular fractionation from WT mouse brains and Parkin overexpressing (OE) M17 cells. The identity and purity of each fraction were confirmed by organelle-specific markers, including Sigma 1 Receptor (Sigma1R) for ER and MAMs, VDAC1 for both mitochondrial and MAMs, and cytochrome c oxidase subunit IV (COX IV) for mitochondria 27. As anticipated, endogenous Parkin was present in the purified MAMs fractions in WT mouse brain and Parkin OE M17 cells (Fig. 3A-B).

Next, IP and MS analyses were adopted to explore potential working partners of Parkin in the MAMs, with samples derived from substantia nigra of mouse brain. MS results revealed that among all known MAMs tethering proteins, Parkin interacted with VDAC1, Grp75 (also called HSPA9), ITPR1 (also called IP3R) and MFN2 (Fig. S3A-B). As previously mentioned, the IP3R-Grp75-VDAC1 complex is one of the four MAMs tethering complexes and the direct calcium flux channel in mammalian cells 31, 32. Therefore, the interaction between Parkin and this tripartite complex was screened by PLA. The results revealed clear colocalization of Parkin with IP3R, Grp75, and VDAC1 in control M17 cells (Fig. 3C). As negative controls, no PLA signals were observed with the same combinations in Parkin KO cells, and likewise, no signals were detected in normal M17 cells with these primary antibodies paired with IgG controls (Fig. S3C-J). Co-IP analysis also confirmed these interactions in Parkin OE M17 cells (Fig. 3D). These interactions were also validated in mouse brain (Fig. 3E). We extracted ER fractions and found no interaction signals between Parkin and IP3R on the ER (Fig. S3K). Importantly, IP3R, Grp75, and VDAC1 interacted with Parkin in the MAMs fraction of mouse brain (Fig. 3F), suggesting that this interaction occurs in ER-mitochondria contact sites under physiological conditions.

Blue native-polyacrylamide gel electrophoresis (BN-PAGE) was conducted on CM preparations, including both mitochondria and MAMs, to determine whether Parkin is a native component of the IP3R-Grp75-VDAC1 complex. A large protein complex of the IP3R-Grp75-VDAC1 complex was observed on BN-PAGE as previously reported 32, 33. Interestingly, Parkin was detected in the large complex (Fig. 3G). This result was also validated in the CM fractions prepared from mouse brains (Fig. 3H). Furthermore, 2-dimensional sodium dodecyl-sulfate polyacrylamide gel electrophoresis (2D SDS-PAGE) analysis of Parkin OE M17 cells distinctly revealed the presence of IP3R, Grp75, and VDAC1 along with Parkin in this complex (Fig. 3I). Collectively, these findings demonstrate that Parkin physically interacts with the IP3R-Grp75-VDAC1 complex in the MAMs.

### Calcium-dependent localization of Parkin at the MAMs

**Figure 4:**
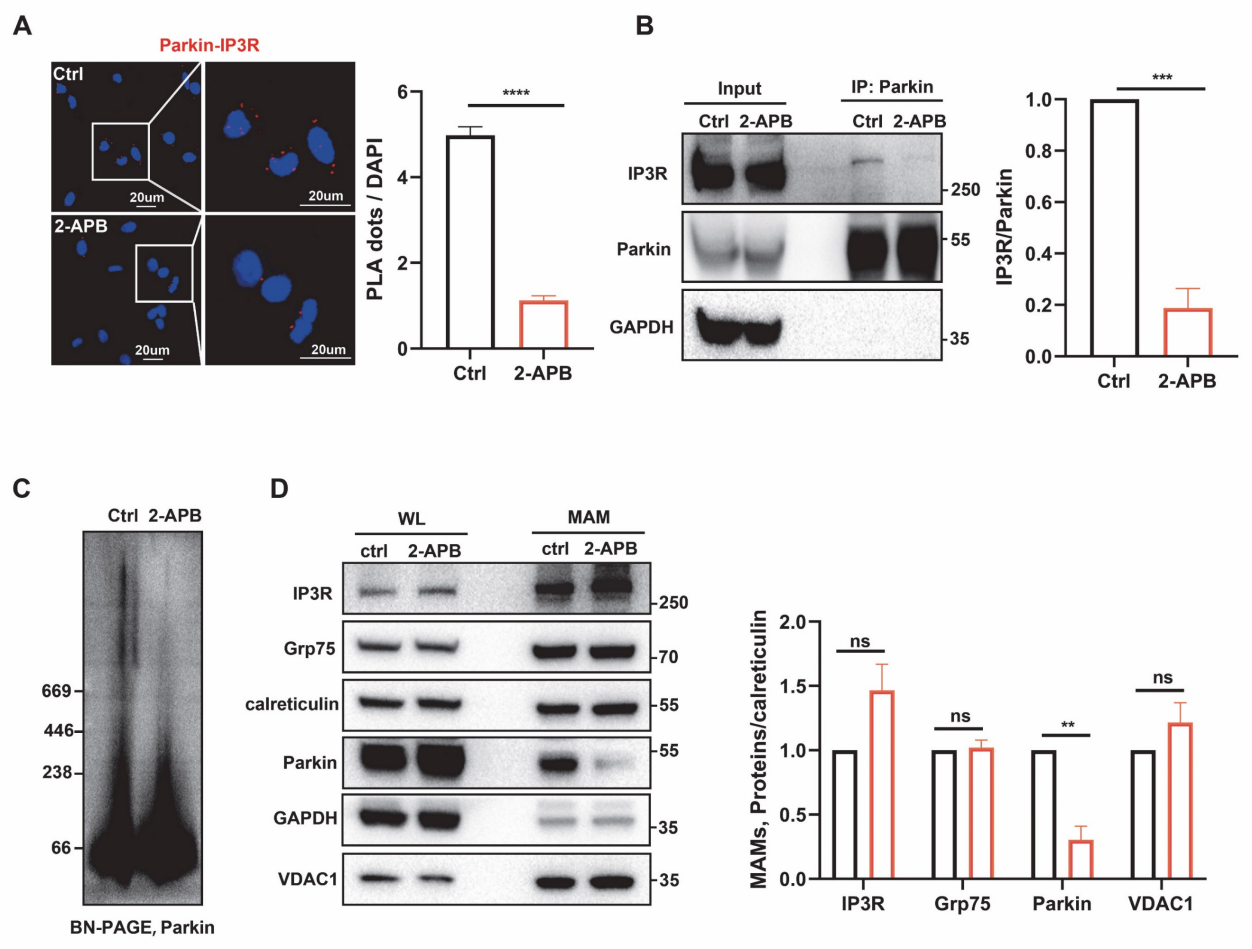
** Calcium-Dependent Localization of Parkin at the MAM.** (A) Representative images of PLA targeting IP3R-Parkin interactions after treatment with 2-APB (50 μm, 2 hours) in M17 cells (Scale bar, 20 μm). (B) Representative immunoblot and quantitative analyses of IP3R coimmunoprecipitated with parkin after treatment with 2-APB (50 μm, 2 hours) in overexpressing WT parkin M17 cells. Three independent experiments were conducted and were quantified. (C) BN-PAGE of the complex after treatment with 2-APB (50 µm, 2 hours). Representative immunoblot images were detected by parkin. Three independent experiments were conducted. (D) Immunoblot analysis of MAM proteins in the WL and MAM fractions from control and 2-APB treatment (50 µm, 2 hours). Proteins were normalized to calreticulin. Three independent experiments were conducted and were quantified. Data are expressed as means ± SEM based on three independent experiments. Data were analyzed using a two-tailed unpaired Student's t-test. *P < 0.05; **P<0.01; ***P < 0.001.

Given the physiological localization of Parkin at the MAMs and its interaction with the IP3R complex, we further explored the mechanisms underlying Parkin recruitment. PINK1 is known to recruit Parkin during mitophagy. However, we found that under physiological conditions, PINK1 KO did not affect the interaction between Parkin and IP3R (Fig. S4A-B), possibly because PINK1 is cleaved by PARL (Presenilin-associated, rhomboid-like) and degraded by the proteasome, resulting in low cytoplasmic levels and being undetectable at the MAMs 8, 15, 34. Recent research also indicates that Parkin localization can be independently regulated by Ca^2+^ signaling 35, 36. When we blocked IP3R-mediated calcium influx using 2-Aminoethoxydiphenyl borate (2-APB) 37, the interaction signal was significantly reduced (Fig. 4A). In addition, co-IP further validated this finding, showing a significant reduction in the interaction between Parkin and IP3R following 2-APB treatment (Fig. 4B). We then utilized BN-PAGE to assess Parkin's localization within complexes. The results revealed that blocking calcium flow led to a marked decrease in Parkin's presence within high-molecular-weight complexes (Fig. 4C). Finally, we extracted MAMs fractions and found that blocking calcium flow led to a reduction of Parkin in the MAMs region (Fig. 4D). Based on the above experimental results, we suggest that under physiological conditions, Parkin's localization at the MAMs depends on calcium signaling.

### Parkin regulates the stability of the IP3R-Grp75-VDAC1 complex

**Figure 5:**
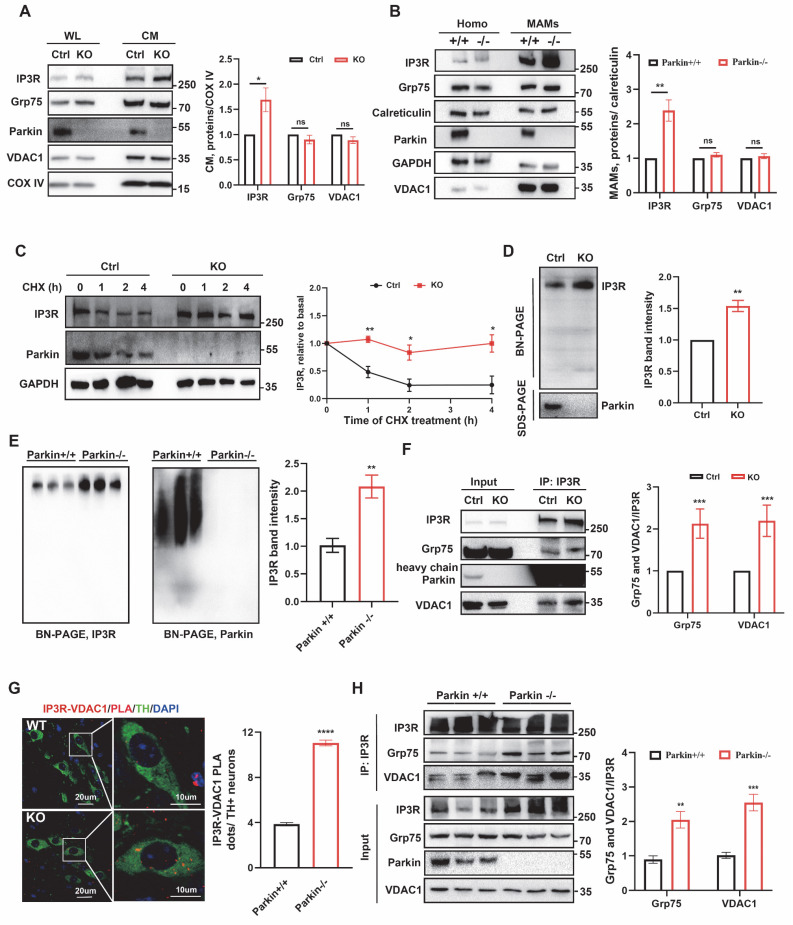
** Parkin regulates the stability of the IP3R-Grp75-VDAC1 complex.** (A) Immunoblot analysis of chosen proteins in control and Parkin KO M17 cells. Control and Parkin KO cells were fractionated into WL and CM. Cytochrome c oxidase subunit IV (COX IV) was used as a loading control for CM proteins. Three independent experiments were conducted and were quantified. (B) Immunoblot analysis of MAM proteins in the WL and MAM fractions from WT and Parkin KO mice (n = 6/group). Proteins were normalized to calreticulin. (C) Immunoblot analyses of time-dependent degradation of IP3R were detected after treatment with cycloheximide (CHX, 100 μg/ml, 0-4 hours) in control and Parkin KO M17 cells. Three independent experiments were conducted and were quantified. (D) BN-PAGE analysis of the macrocomplex detected by IP3R in crude mitochondria from control and Parkin KO M17 cells. SDS-PAGE immunoblot was used to analyze parkin in CM. Data are quantified from three independent experiments. (E) BN-PAGE and immunoblot analysis of the macrocomplex detected by IP3R in the crude mitochondria fraction from mouse brain homogenates (left). Representative immunoblot images detected by parkin (n = 5/group, right). (F) Immunoblot analysis of Grp75/VDAC1 was coimmunoprecipitated with IP3R antibody in control and Parkin KO M17 cells. Three independent experiments were conducted and were quantified. (G) Representative images and quantification of IP3R-VDAC1 PLA signals revealed an association in tyrosine hydroxylase positive (TH+) neurons in the substantia nigra of Parkin KO mice. IP3R-VDAC1 PLA (red) was performed in brain sections and co-stained with TH (green) (n = 5/group), (Scale bar, 20 µm). (H) Immunoblot analysis of Grp75 and VDAC1 was coimmunoprecipitated with IP3R antibody in the brain homogenates of wild-type control and Parkin KO mice (n = 5/group). Data are expressed as means ± SEM. Data were analyzed using two-tailed unpaired Student's t-test; *P < 0.05; **P < 0.01; ***P < 0.001.

We investigated the effect of Parkin on the IP3R-Grp75-VDAC1 tethering complex in MAMs. Compared with control cells, Grp75 and VDAC1 levels were unchanged in whole-cell lysates (WL) and CM fractions of Parkin KO M17 and SH-SY5Y cells. In contrast, IP3R expression was markedly elevated in both WL and CM fractions of Parkin KO cells (Fig. 5A and Fig. S5A), consistent with increased IP3R levels in WL and MAM fractions of Parkin KO mouse brain (Fig. 5B). Real-time PCR revealed no significant changes in IP3R mRNA *in vitro* or *in vivo*, indicating that Parkin loss does not affect transcription (Fig. S5B-C). These findings, together with Parkin's E3 ubiquitin ligase activity, suggest that the IP3R accumulation may result from impaired degradation. Cycloheximide (CHX) chase experiments showed that in Parkin OE M17 cells, ~50% of IP3R was degraded within 2 hours, whereas Grp75 and VDAC1 levels remained largely stable (Fig. S5D). This rapid degradation was blocked in Parkin KO M17 cells (Fig. 5C), suggesting that Parkin may selectively mediate fast IP3R turnover to dynamically regulate calcium homeostasis, without substantially affecting Grp75 or VDAC1.

Furthermore, we investigated the effect of Parkin on the IP3R-Grp75-VDAC1 complex. BN-PAGE analysis showed that the IP3R complex was dramatically elevated in Parkin KO M17 cells, and a similar elevation was observed in Parkin KO SH-SY5Y cells (Fig. 5D and Fig. S5E). The IP3R complex was also similar in ventral midbrain lysates from WT and Parkin KO mouse brain (Fig. 5E). Notably, both the protein homeostasis of IP3R and its interactions with other components are pivotal for the IP3R-Grp75-VDAC1 complex. As shown in Fig. 1B, IP3R/VDAC1 PLA signals increased substantially in Parkin KO cells. Consistently, quantitative Co-IP analysis showed that significantly higher amounts of Grp75 and VDAC1 were pulled down by IP3R in the Parkin KO M17 cells, with similar results observed in Parkin KO SH-SY5Y cells (Fig. 5F and Fig. S5F). Subsequently, *in situ* IP3R-VDAC1 PLA experiments were performed on mouse brain sections to further evaluate the IP3R-Grp75-VDAC1 complex and MAMs structure. Tyrosine hydroxylase (TH) was used to label DA neurons, which were co-stained. IP3R-VDAC1 PLA signals in TH neurons were calculated. Parkin KO TH neurons exhibited heightened IP3R/VDAC PLA signals (Fig. 5G). These enhanced interactions were also validated in ventral midbrain lysates from WT and Parkin KO mouse brain by quantitative co-IP analysis (Fig. 5H). Collectively, these results demonstrate that the integrity of the IP3R-Grp75-VDAC1 complex is modulated by Parkin both *in vitro* and *in vivo*.

### Parkin tunes the degradation of IP3R through K48-linked ubiquitination

**Figure 6:**
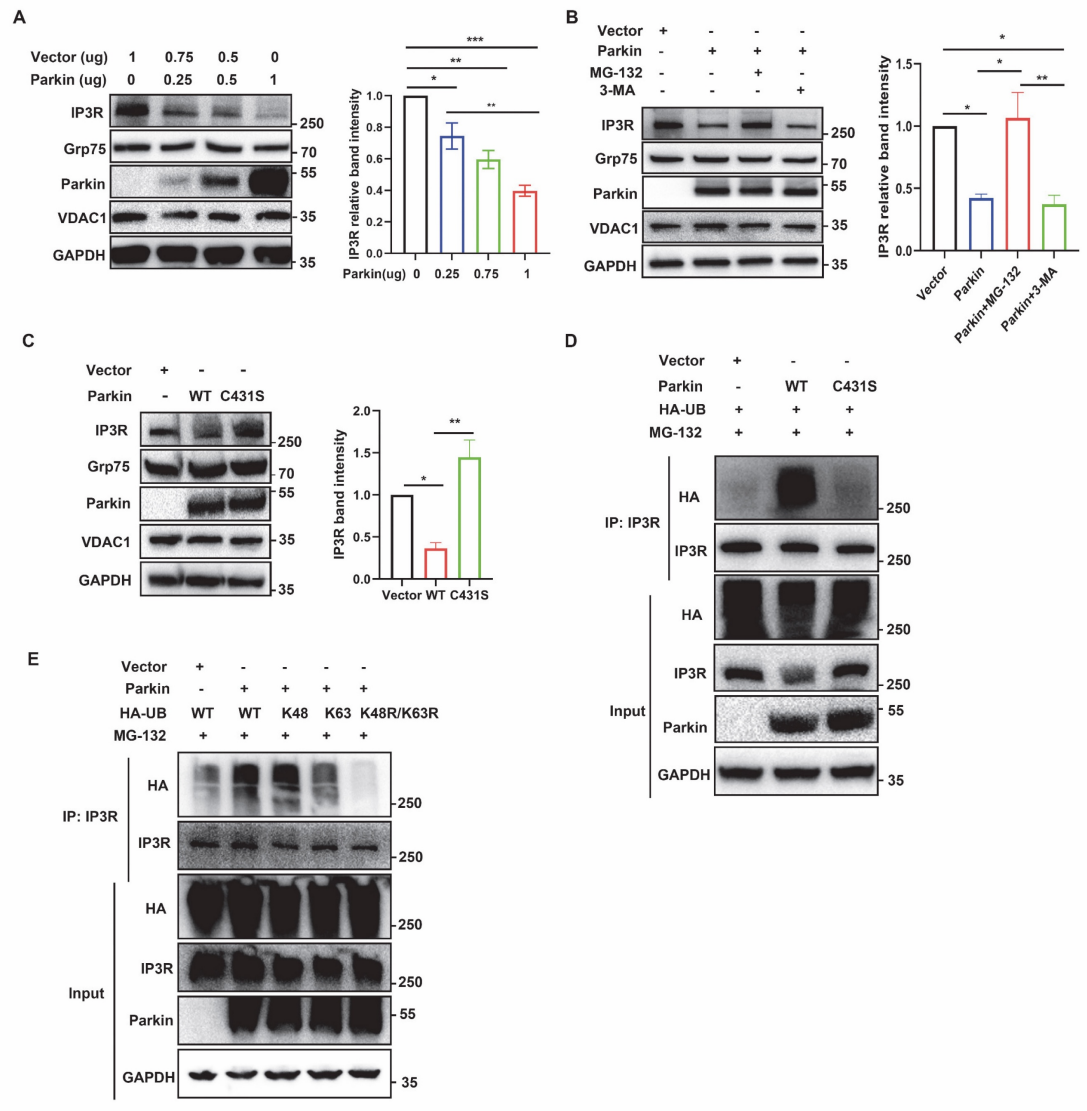
** Parkin tunes the degradation of IP3R through K48-linked ubiquitination.** (A) Immunoblot analyses of IP3R protein showed its reduced stability by Parkin-dependent expression in M17 cells. Three independent experiments were conducted and were quantified. (B) Immunoblot analysis was performed to assess IP3R protein expression in M17 cells overexpressing wild-type (WT) parkin, treated with MG132 (20 µM) or 3-MA (2.5 mM) for 24 hours. Three independent experiments were conducted and were quantified. (C) Representative immunoblot and quantitative analyses of the degradation of IP3R from overexpressing vector, WT parkin, and parkin C431S mutant M17 cells. Three independent experiments were conducted and were quantified. (D) Ubiquitination levels of IP3R were assessed in M17 cells expressing WT or C431S parkin plasmid. Cells were treated with 20 µM of MG132 for 4 hours. Three independent experiments were conducted. (E) Ubiquitination levels of IP3R were assessed in M17 cells expressing wild-type (WT) parkin, HA-Ub, and its mutant plasmids. Cells were treated with 20 µM of MG132 for 4 hours. Three independent experiments were conducted. Data are expressed as means ± SEM based on three independent experiments. Data were analyzed using ANOVA with Tukey's multiple comparisons test (A-C). *P < 0.05, **P < 0.01, ***P < 0.001.

To investigate the regulatory mechanism of IP3R by Parkin, we further examined cellular responses upon gain of Parkin function. Accompanied by increasing Parkin overexpression, IP3R levels gradually decreased (Fig. 6A). The proteasome inhibitor MG-132 and the autophagy-lysosome inhibitor 3-MA were used to determine the degradation pathway of IP3R. The decreased IP3R levels after Parkin overexpression was reversed by adding MG132 but were not elevated after 3-MA treatment, indicating that Parkin reduces IP3R stability through the proteasome pathway (Fig. 6B). We also examined IP3R levels in the presence of WT Parkin or an E3 activity-dead Parkin C431S 38. As expected, overexpression of WT Parkin significantly reduced the IP3R level; this effect was suppressed by Parkin C431S (Fig. 6C). Moreover, expression of the pathogenic RING-domain mutants T240R and R275W also prevented IP3R degradation (Fig. S6A), indicating that Parkin-mediated IP3R turnover depends on its functional E3 ubiquitin ligase activity.

Under basal conditions, ubiquitination assays showed that IP3R was ubiquitinated by WT Parkin, as indicated by a smear of HA-Ub signal, whereas this effect was abolished by the catalytically inactive mutant Parkin C431S (Fig. 6D). In three independent experiments, Parkin OE M17 cells were transfected with HA-Ub and treated with MG-132 for 6 h to enrich ubiquitinated proteins, enabling identification of Parkin-mediated ubiquitination sites on IP3R. MS detected peptides covering ~85% of the IP3R sequence, including 155 of its 167 lysines (Fig. S6B), indicating no regional bias in ubiquitination detection.

IP3R undergoes ubiquitination mainly through K48- and K63-linked chains. To further elucidate the characteristics of Parkin-mediated IP3R ubiquitination, we determined the types of ubiquitin linkage using K48-specific and K63-specific ubiquitin plasmids in which only one specific lysine was retained. The ubiquitination assays revealed that IP3R was extensively ubiquitinated with Ub-K48 but not Ub-K63 (Fig. 6E). Using mutant Ub-K48R/K63R, we found that the polyubiquitination of IP3R was abolished (Fig. 6E).

### IP3R is ubiquitinated by Parkin upon induction of E3 activities

**Figure 7:**
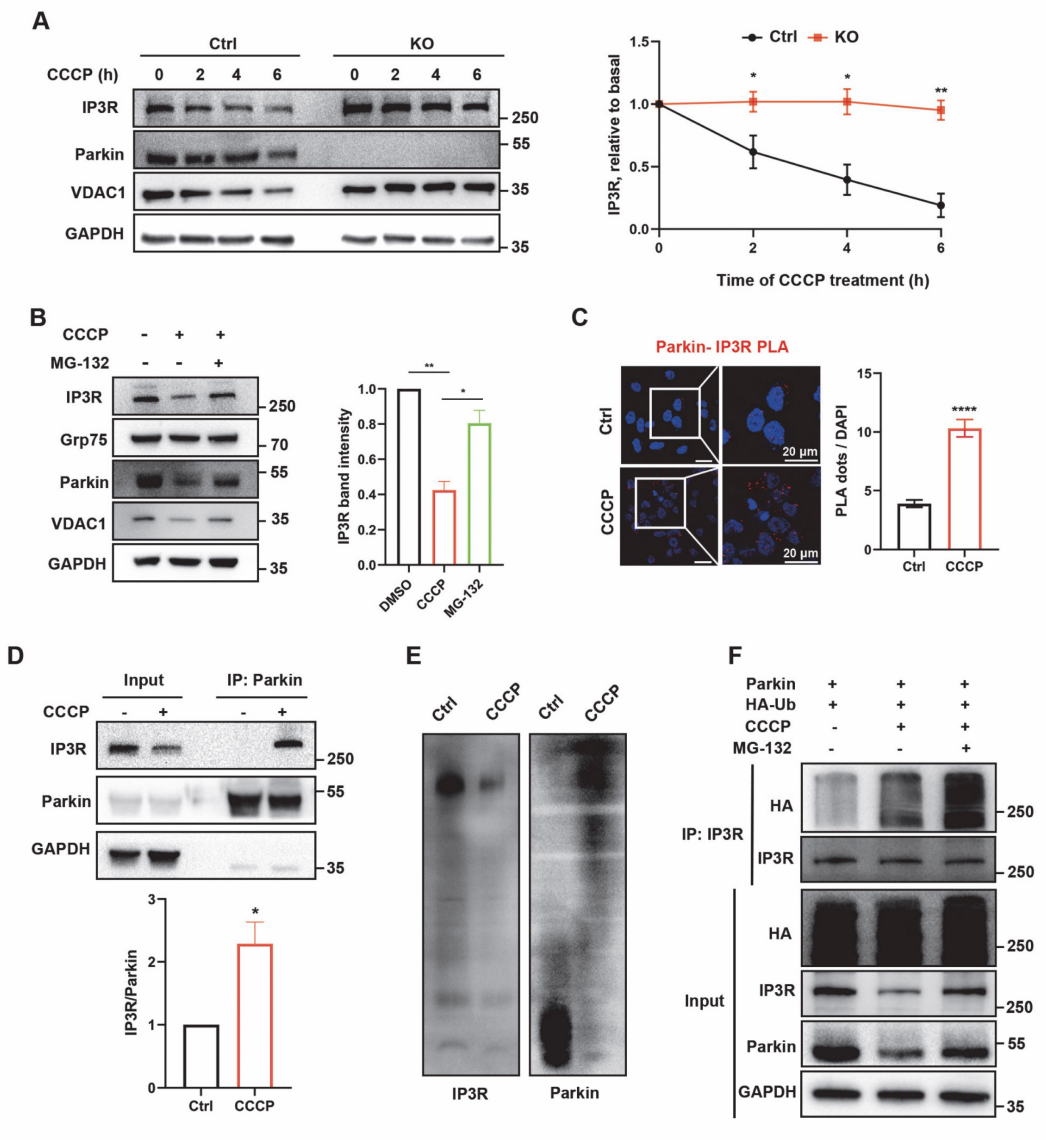
** IP3R is ubiquitinated by Parkin upon induction of E3 activities.** (A) Immunoblot analyses of time-dependent degradation of IP3R were detected after treatment with carbonyl cyanide m-chlorophenyl hydrazine (CCCP, 20 µm, 0-6 hours) in control and Parkin KO M17 cells. Three independent experiments were conducted and were quantified. (B) Representative immunoblot and quantitative analyses of IP3R degradation after treatment with CCCP (20 µm, 2 hours) and proteasome inhibitor MG-132 (20 µm, 0.5 hour before CCCP treatment) in M17 cells. Three independent experiments were conducted and were quantified. (C) Representative images of PLA targeting IP3R-Parkin interactions after treatment with CCCP (20 µm, 1 hour) in M17 cells (n = 166-199 cells). (D) Representative immunoblot and quantitative analyses of IP3R coimmunoprecipitated with parkin after treatment with CCCP (20 µm, 1 hour) in overexpressing WT parkin M17 cells. Three independent experiments were conducted and were quantified. (E) BN-PAGE of the complex after treatment with CCCP (20 µm, 2 hours). Representative immunoblot images were detected by IP3R (left) and parkin (right). Three independent experiments were conducted. (F) Ubiquitination levels of IP3R after treatment with CCCP (20 µm, 2 hours) and MG-132 (20 µm, 1 hour before CCCP treatment) in M17 cells. Three independent experiments were conducted. Data are expressed as means ± SEM from three independent experiments. Data were analyzed using a two-tailed unpaired Student's t-test (A, C-F) and ANOVA with Tukey's multiple comparisons test (B). *P < 0.05; **P < 0.01; ***P < 0.0001.

Parkin E3 activities can be activated by mitochondrial uncoupler carbonyl cyanide m-chlorophenyl hydrazine (CCCP). After such stimulation, Parkin causes proteasomal degradation of various outer mitochondrial membrane protein (35). CCCP treatment led to a time-dependent reduction of IP3R in control cells, whereas IP3R levels remained unchanged in Parkin KO cells (Fig. 7A). Consistently, CCCP treatment reduced IP3R levels, which were remarkably blocked after MG-132 treatment (Fig. 7B). Subsequently, colocalization analysis was performed to further investigate whether Parkin is recruited to IP3R. IP3R/Parkin PLA signals significantly increased after CCCP treatment (Fig. 7C), indicating Parkin recruitment to IP3R. CCCP-induced Parkin recruitment was also observed via Co-IP assays (Fig. 7D). Additionally, BN-PAGE analysis revealed that the majority of lower Parkin complexes translocated upward to the vicinity of the IP3R macrocomplex, which was decreased after CCCP treatment (Fig. 7E). Moreover, the ubiquitination modification of IP3R was distinct after CCCP and MG-132 treatment (Fig. 7G). Altogether, these results indicate that Parkin in the MAMs ubiquitinates IP3R for degradation to maintain its protein homeostasis. Overall, these data suggested that Parkin directly counter IP3R-mediated mitochondria-ER tethering through IP3R turnover to promote mitophagy.

## Discussion

**Figure 8:**
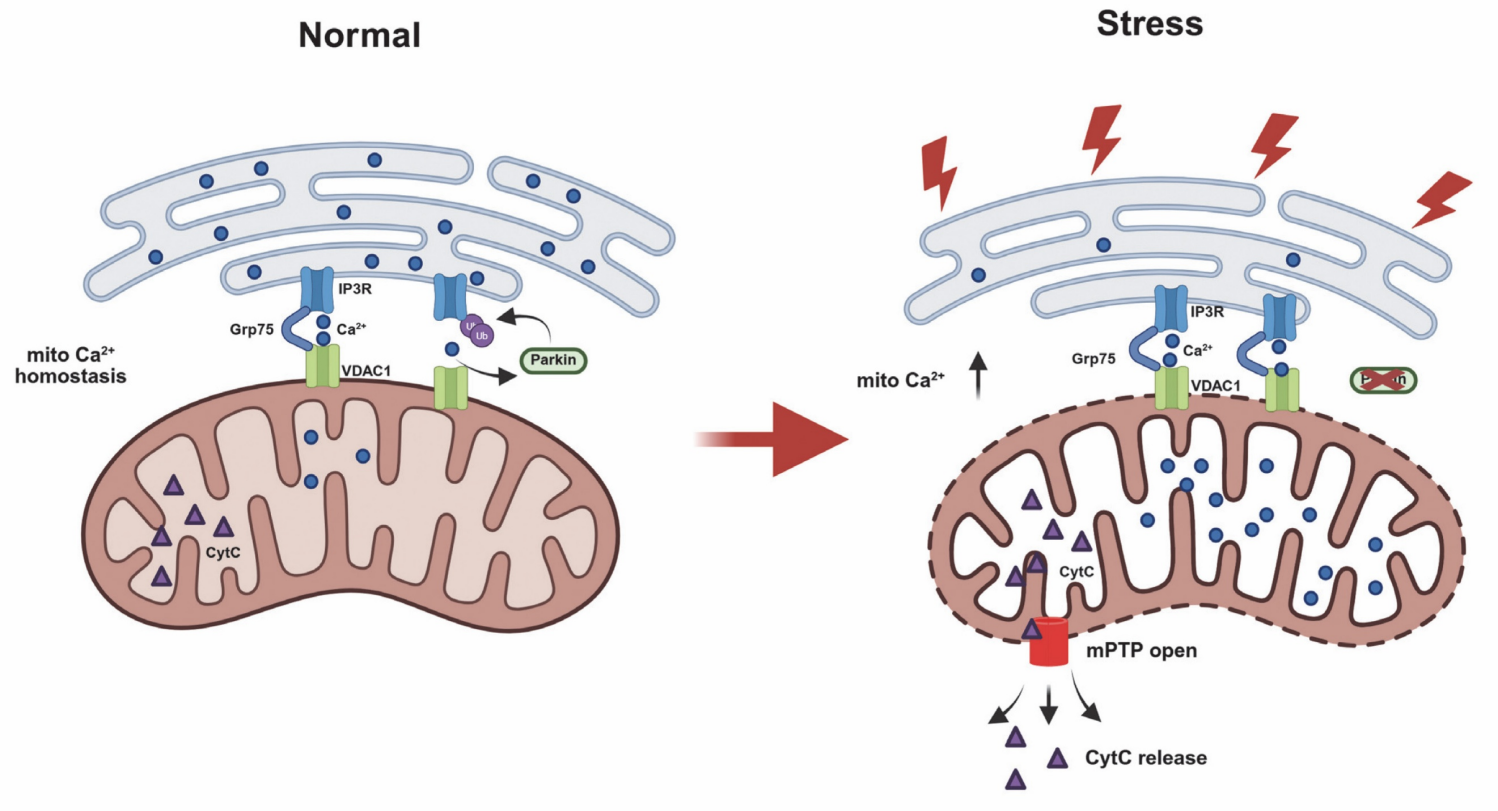
** Schematic diagram illustrating Parkin-mediated regulation of ER-mitochondria contacts and calcium homeostasis.** Under normal conditions, IP3R-mediated Ca²⁺ flux recruits Parkin. Through its E3 ubiquitin ligase activity, Parkin ubiquitinates and degrades IP3R channels. This process leads to disassembly of the IP3R complex and helps regulate mitochondrial calcium homeostasis in mitochondria-associated membranes (MAMs). In the absence of functional Parkin, IP3R complexes abnormally accumulate at MAM regions. This increases cellular sensitivity to calcium stimuli and leads to mitochondrial calcium overload, triggering abnormal opening of the mitochondrial permeability transition pore (mPTP) and ultimately resulting in apoptosis.

This study reveals a newly identified role of Parkin in regulating ER-mitochondria calcium homeostasis via modulation of the IP3R-Grp75-VDAC1 complex. Parkin is recruited to MAMs through IP3R-dependent Ca²⁺ flux, where it ubiquitinates IP3R to promote its degradation. Loss of Parkin impairs this regulation, resulting in IP3R accumulation at MAMs, excessive mitochondrial calcium uptake, mPTP opening, and heightened cellular vulnerability to stress. (Fig.8).

MAMs are key hotspot areas for the regulation of calcium communication between mitochondria and ER and may play an important role in PD pathogenesis 5, 6. PINK1/Parkin has also been found to be localized at ER-mitochondria contact sites and modulate mitophagy in this structure 39. However, the role of Parkin at the MAMs remains controversial, perhaps owing to different detection techniques and quantification methods 40, 41. Fluorescence-based detection methods assess ER-mitochondria interactions, but cannot accurately quantify MAMs due to fluorescence signal specificity and limited precision at 100 nm 5. Given the limitations of fluorescence resolution, PLA can detect interactions at distances of less than 40 nm 29. The resolution advantage of PLA can overcome this issue. By quantifying the interactions of complex proteins, it can indirectly reflect the increase or decrease of MAMs. Our results showed that PLA revealed enhanced associations between the ER and mitochondria in Parkin KO cells. Importantly, PLA also demonstrated similar changes in ER-mitochondria interactions in DA neurons of the mouse substantia nigra. EM is considered the gold standard for detecting MAMs 6. However, previous studies have employed inconsistent or non-standardized metrics for measuring MAMs in electron microscopy. Some studies have only reported the average distance between the mitochondria and the ER, while others have quantified the number of ER structures within a 500 nm radius of mitochondria. According to the definition of MAMs, which is the area where the distance between the ER and mitochondria is less than 30 nm, these indicators do not accurately reflect MAMs 40, 41. According to the EM definition of MAMs, we calculated the MAMs coverage, and the average length of these regions 10. We found that, both *in vivo* and *in vitro*, Parkin KO led to increased MAMs coverage and MAMs length. Using EM, Mclelland *et al.* reported that Parkin deletion impaired the detethering of mitochondria from the ER during mitophagy 42. Grossmann *et al.* also showed that Parkin deficiency in iPSC-derived DA neurons increased ER-mitochondria associations by split fluorescence reporters 43. Thus, these results collectively demonstrate that Parkin deficiency enhance ER-mitochondria associations in DA neurons.

Approximately 80% of mitochondrial calcium is regulated by MAMs, so an increase in MAMs may lead to mitochondrial calcium imbalance, resulting in reduced ATP production, increased mPTP opening, and ultimately, cell apoptosis 23, 24. Previous reports have reported that Parkin deficiency disrupts intracellular calcium homeostasis (43-45). Our data demonstrated that Parkin KO led to a mild increase in mitochondrial calcium levels, and caused a slight opening of the mPTP. More importantly, Parkin KO M17 cells were more susceptible to calcium stimulants (e.g., TG), exhibiting increased ER calcium release and elevated mitochondrial calcium levels, which subsequently led to mPTP channel opening and ultimately resulted in cell apoptosis. DA neurons are autonomous pacemakers with dynamic calcium signaling 44, which may underlie the heightened vulnerability of DA neurons following Parkin KO. Taken together, these data underscore the role of Parkin in calcium homeostasis; however, the exact mechanisms are yet to be understood.

Our study confirmed that endogenous Parkin is partially located at the MAMs fraction. MS results suggested that under physiological conditions, Parkin interacted with the IP3R-Grp75-VDAC1 complex. Prior research has shown that Parkin targets various mitochondrial substrates for ubiquitination 16. This process is mediated by the PINK1/Parkin-dependent mitophagy pathway and may involve the degradation of MAMs proteins such as MFN2, VDACs, and Miro 45-47. Some MAMs proteins primarily interact with Parkin during mitophagy, and therefore are not detected by MS under physiological conditions. Through co-IP experiments with different organelle fractions, we found that Parkin interacts with the complex in the MAMs region, rather than the ER. BN-PAGE further confirmed the localization of Parkin on the complex under physiological conditions. Under physiological conditions in M17 cells, most Parkin remains in a resting state, with only a small fraction activated to interact with the IP3R complex. This is reflected in BN-PAGE as a minor portion of Parkin appearing in high molecular weight complexes. Parkin recruitment can be triggered by two distinct mechanisms. On one hand, when the mitochondrial membrane potential is disrupted by agents such as CCCP, FCCP or antimycin/oligomycin (A/O), PINK1 accumulates on the outer mitochondrial membrane, where it promotes Parkin translocation and mitochondrial recruitment 19. Under CCCP-induced mitophagy, Parkin shows increased colocalization with the IP3R complex and redistributes into high molecular weight complexes, reflecting its recruitment to the mitochondrial outer membrane where it ubiquitinates multiple outer membrane and MAM-associated proteins 16, 48. This extensive ubiquitination increases the apparent molecular weight of the complexes, leading to their shift to higher molecular weight regions on BN-PAGE 49. On the other hand, calcium elevation can also promote Parkin recruitment 36, 50. Calcium-dependent factors, including calcineurin in the cytosol and Miro1 on mitochondria, have been implicated in Parkin recruitment 35, 36. Compared with PINK1-mediated translocation, calcium-dependent recruitment of Parkin is relatively weak but may better represent physiological regulation. In our study, this process was independent of PINK1 and instead relied on IP3R-mediated calcium signaling, consistent with this notion. We also found that Parkin distribution in native complexes of the mouse brain differs from that in M17 cells, being almost entirely concentrated in medium and high molecular weight regions. As mentioned above, this pattern in neurons may result from frequent calcium flux and enhanced mitophagy driven by high oxidative phosphorylation (OXPHOS) activity, both of which likely contribute to the altered distribution of Parkin in the brain.

The current study also uncovered that IP3R may serve as the target for Parkin in regulating ER-mitochondria association. IP3R is prone to rapid degradation through ubiquitination to achieve protein homeostasis and suitable calcium signaling 51, 52. Calcium release from the ER via IP3R channels leads to more conformational changes and opening of these channels, which rapidly creates a calcium hotspot that triggers mitochondrial calcium uptake 53. More importantly, along with the calcium-induced IP3R conformational change, the buried ubiquitination residues of IP3R are exposed to degradation 54, 55. Our previous study showed that another PD-associated protein DJ-1 regulates IP3R stability and function. DJ-1 deficiency leads to the accumulation of the IP3R protein, which negatively impacts the functionality of the complex, resulting in the disassembly of the IP3R-Grp75-VDAC1 complex 10. Contrary to DJ-1 deficiency, Parkin deficiency primarily increases functionally active IP3R. Specifically, the interaction between IP3R, VDAC1, and Grp75 increases with an increase in the abundance of IP3R in the native complex. Nonetheless, the underlying mechanism remains unknown, probably due to the spatial conformation of IP3R. In addition to its involvement in the stabilization of the IP3R-Grp75-VDAC1 complex, DJ-1 may also help induce spatial conformational changes in IP3R, thereby participating in calcium transport and ubiquitin-mediated degradation. Meanwhile, Parkin primarily exerts its role through E3 ubiquitin ligase activity for ubiquitination and degradation. Whether these two proteins collaborate to regulate the IP3R complex during this process remains mysterious, warranting further exploration in future studies. Interestingly, evidence from recent studies have corroborated our findings. Ham *et al.* reported that PINK1/Parkin deficiency results in enhanced ER calcium release and cytoplasmic calcium overload. Mechanistically, PINK1/Parkin indirectly regulates IP3R channel activity through CISD1, modulating ER-to-cytosol calcium flux 56. Our study shows that Parkin directly regulates IP3R channels at MAMs and disassembles the IP3R-Grp75-VDAC1 complex, ensuring proper mitochondrial calcium levels. Together with the findings of Ham *et al.*, these results highlight Parkin's key role in maintaining cellular calcium homeostasis, both cytosolic and mitochondrial, suggesting it as a potential therapeutic target for PD. Inhibitors of calcium-regulating proteins could rescue this process and serve as a therapeutic intervention for Parkin-deficient PD. Previous studies have also shown that inhibiting mitochondrial calcium transport proteins, such as the mitochondrial calcium uniporter and VDAC1, can partially rescue Parkin-deficient PD 57. The development of therapeutic targets and small molecule drugs in this area warrants further investigation in the future.

## Conclusion

In summary, our study reveals that Parkin is involved not only in mitophagy but also in the regulation of MAMs under physiological conditions. Specifically, under physiological conditions, Parkin is recruited by IP3R-mediated calcium flow and participates in the regulation of calcium homeostasis, especially in neurons where calcium ion activity is particularly frequent. This may be an important mechanism by which Parkin deficiency contributes to the pathogenesis of PD.

## Supplementary Material

Supplementary figures.

## Figures and Tables

**Table 1:** Primer sequences for RT-qPCR

Gene	Sample	Forward	Reverse
IP3R	M17 cells	TCTCAGACCAGAGTACGACTT	CAGACAGCACCCGAATACAG
Actin	M17 cells	AGAGCTACGAGCTGCCTGAC	AGCACTGTGTTGGCGTACAG
IP3R	Mouse	CGATGACATCGTTCGTGTGGTC	CACCTCCGTATCCACATAGCAG
GAPDH	Mouse	GGTTGTCTCCTGCGACTTCA	TGGTCCAGGGTTTCTTACTCC

## Abbreviations

PD: Parkinson's disease
DA: dopaminergic
PINK1: PTEN-induced kinase 1
ER: endoplasmic reticulum
MAM: mitochondria-associated ER membrane
IP3R: inositol-1,4,5-triphosphate receptor
mPTP: mitochondrial permeability transition pores
iPSC: induced pluripotent stem cell
KO: knockout
PLA: proximity ligation assay
WT: wild-type
Rhod-2 AM: Rhod-2 acetoxymethyl ester
TG: thapsigargin
OE: overexpressing
Sigma1R: Sigma 1 Receptor
COX IV: cytochrome c oxidase subunit IV
IP: immunoprecipitation
MS: Mass Spectrometry
BN-PAGE: Blue native-polyacrylamide gel electrophoresis
CM: crude mitochondria
2-APB: 2-Aminoethoxydiphenyl borate
WL: whole cell lysates
TH: Tyrosine hydroxylase
Ub: ubiquitin
CCCP: uncoupler carbonyl cyanide m-chlorophenyl hydrazine
