# Supplementary material for: Parkin Deficiency Impairs ER-Mitochondria Associations and calcium homeostasis via IP3R-Grp75-VDAC1 Complex
Source: Int J Biol Sci. 2026 Jan 1;22(2):731–49. doi: 10.7150/ijbs.121759 (PMC12781089; doi:10.7150/ijbs.121759)
Supplement: Supplementary file 1 — Supplementary figures. [file ijbsv22p0731s1.pdf]

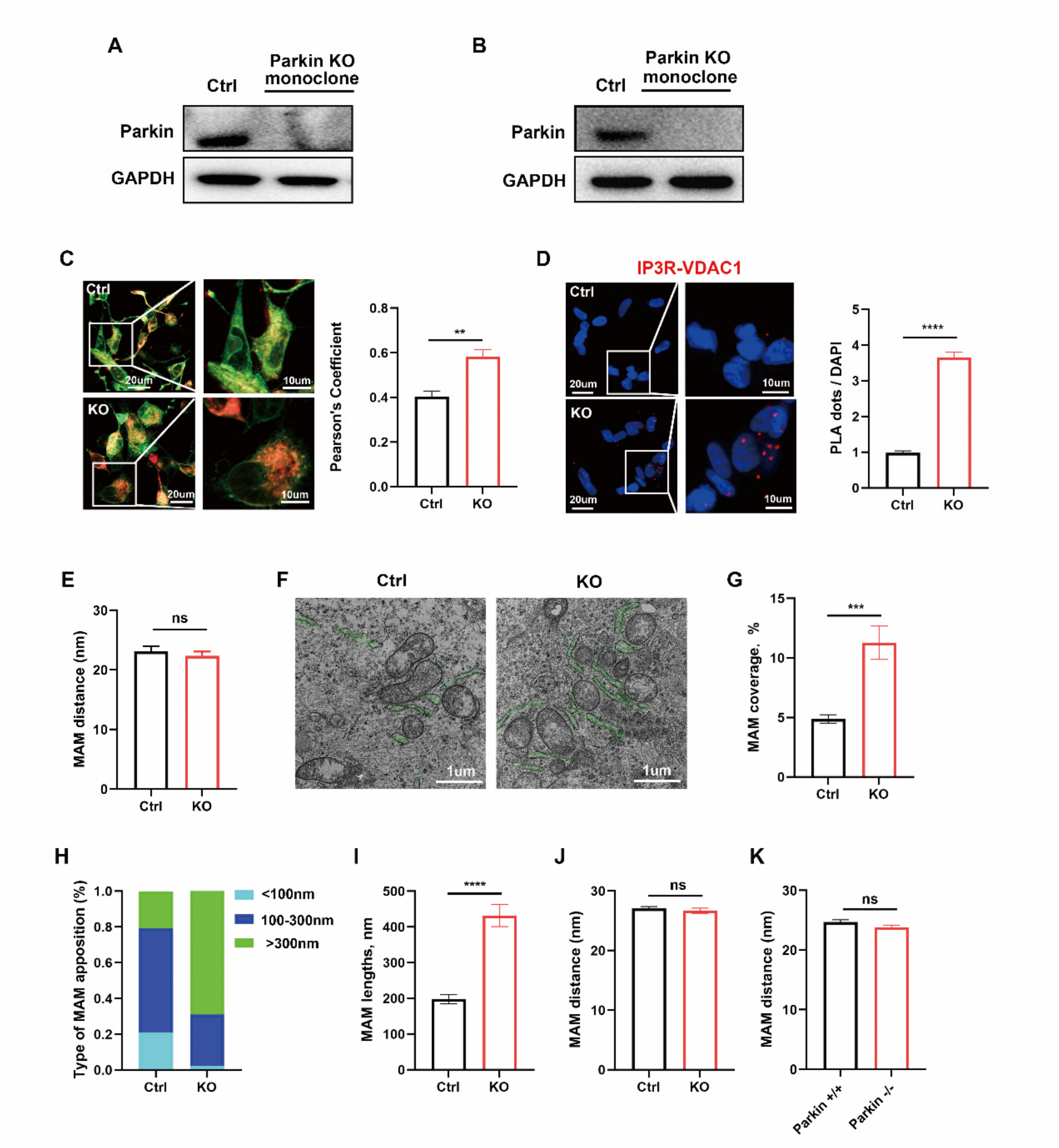

Figure S1 (A and B) Stable Parkin knockout M17 (A) and SH-SY5Y (B) monoclonal line were established using CRISPR/Cas9 gene editing method. (C) Confocal microscopy was used for live cell imaging of both control cells (upper) and Parkin KO SH-SY5Y cells (bottom). ER was stained with an ER-Tracker (green) and mitochondria were stained with MitoTracker (red) (Scale bar, 20 μm). Quantification of ER-mitochondria association was performed using ImageJ (bar graphs on the right). (D) In situ close association between IP3R and VDAC1 was detected by proximity ligation assay (PLA) in control cells (upper) and Parkin KO SH-SY5Y cells (lower) (Scale bar, 20 μm). Quantification is shown in the bar graphs on the right. (E) Quantitative analysis of MAM distance in control (n = 7) and Parkin KO M17 cells (n = 8). (F) Electron micrographs of mitochondria connected to the ER (pseudocolored green) in control and Parkin KO SH-SY5Y cells (Scale bar, 1 μm). (G-I) Quantitative analysis of the percentage of the mitochondria-associated endoplasmic reticulum membrane (MAM) to mitochondria (G), type of MAMs apposition (H) average length (I) and MAM

distance (J) of ER-mitochondria association in control cells (n = 7) and Parkin KO SH-SY5Y cells (n = 7). (K) Quantitative analysis of MAM distance in dopaminergic (DA) neurons from wild-type (WT) and Parkin KO mouse brains (n = 5 mice per group, with 4 neurons per mouse). Data were analyzed using a two-tailed unpaired Student's t-test. *P < 0.05; **P<0.01; ***P < 0.001.

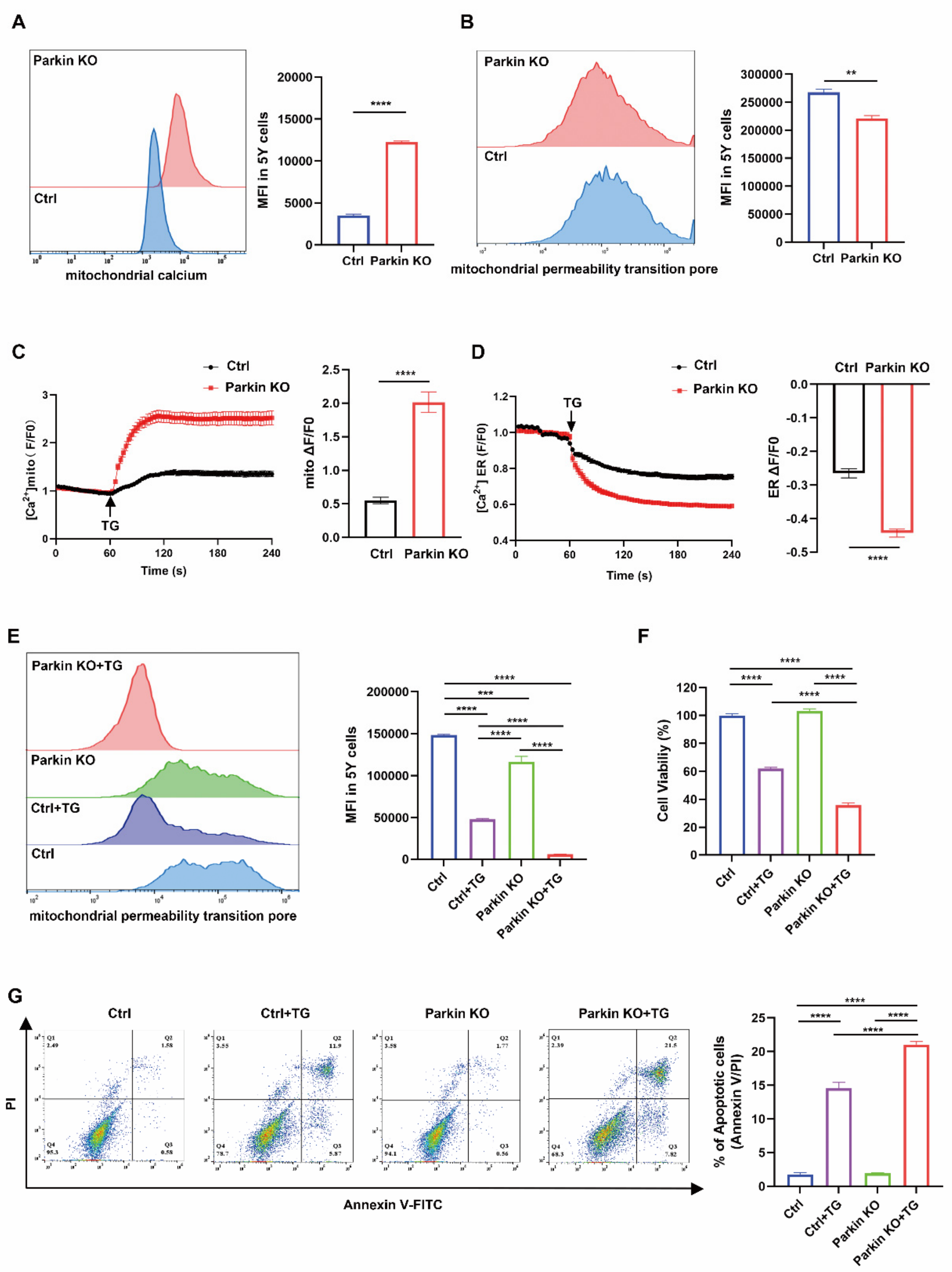

Figure S2 (A) Mitochondrial calcium levels were evaluated using Rhod-2 staining and quantified by mean fluorescence intensity (MFI) through flow cytometry comparisons in control and Parkin KO SH-SY5Y cells. (B) The mPTP functionality was gauged with

calcein-AM staining in conjunction with CoCl2 in control and Parkin KO SH-SY5Y cells. (C and D) Measurement of calcium modulation in mitochondria (C) and the ER (D) in control cells (black) and Parkin KO SH-SY5Y cells (red) was conducted using confocal microscopy. Thapsigargin (TG) was used to initiate calcium release. The right bar graphs indicate the quantification of maximal mitochondrial (C) or ER (D) calcium peak fluorescence during TG treatment (n = 55-65 cells). (E) Treatment with TG (2.5μm, 24 hours) in both genotypes was followed by an analysis of mPTP functionality using calcein-AM fluorescence. (F) CCK-8 assay was used to examine cell viability across four groups after treatment with TG (2.5μm, 24 hours). (G) Apoptosis was analyzed by flow cytometry using Annexin V/PI (propidium iodide) staining, following treatment with TG (2.5 μM, 24 hours). Data are expressed as means ± SEM based on three independent experiments. Data were analyzed using two-tailed unpaired Student's t-test (A-D) and one-way analysis of variance (ANOVA) with Tukey's multiple comparisons test (E-F). *P < 0.05; **P<0.01 ; ***P < 0.001.

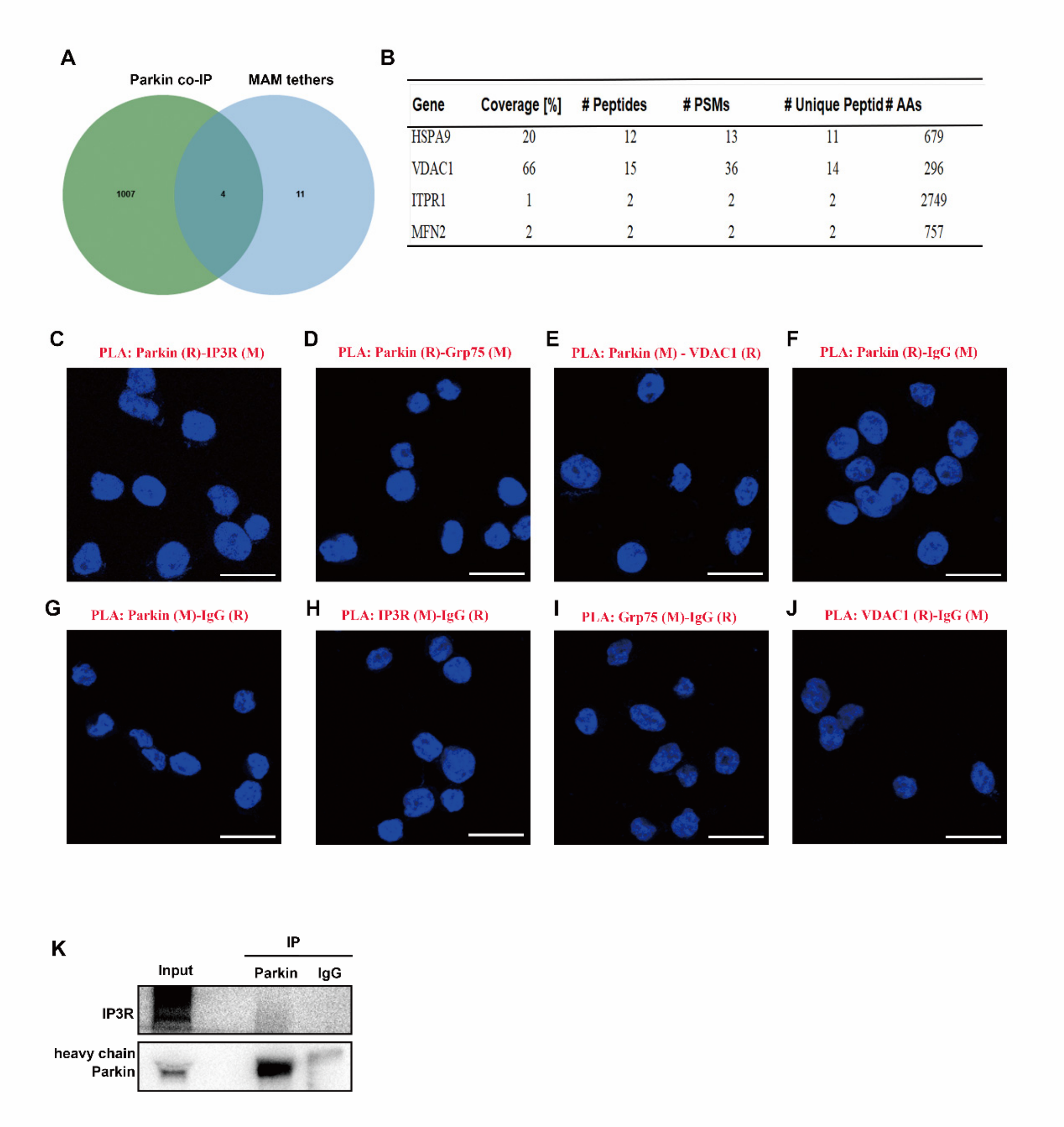

Figure S3 (A)Venn diagrams showed overlap between MAM (mitochondrial-associated

ER membrane) tethers and Parkin interacting proteins as identified by mass spectrometry. (B)The lists of Parkin interacting proteins located in the MAM tethers. (C-E) Biological control experiments using Parkin KO M17 cells were performed to confirm that the observed PLA signals indicating the close proximity of Parkin with IP3R (C), Grp75 (D), and VDAC1 (E) reflect specific interactions rather than non-specific background. (F-J) The technical control experiments of PLA were performed in normal control M17 cells by labelling the indicated proteins with an unspecific IgG from different species to rule out the nonspecific binding of the antibodies used in this study. Scale bar, 20 μm. (K) Immunoblot analysis of IP3R in the parkin immunoprecipitates of ER fractions from parkin-overexpressing M17 cells. Three independent experiments were conducted.

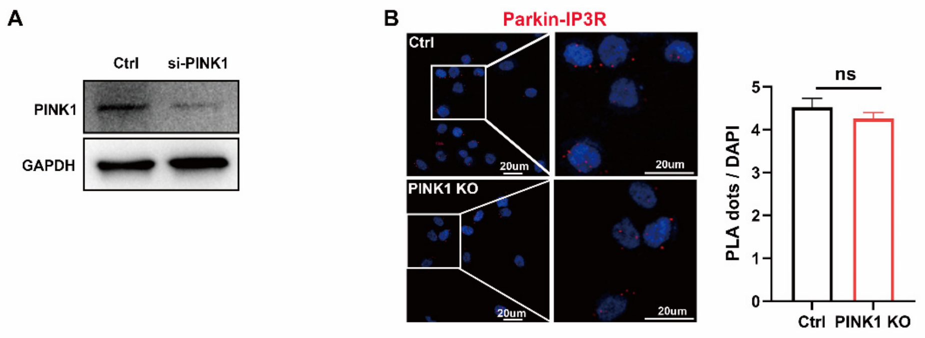

Figure S4 (A) Immunoblot analysis was performed to assess the efficiency of PINK1 siRNA knockdown. Three independent experiments were conducted. (B) Representative images of PLA showing IP3R-Parkin interactions following PINK1 KO in M17 cells (Scale bar, 20 μm). Data were analyzed using a two-tailed unpaired Student's t-test. *P < 0.05; **P<0.01; ***P < 0.001.

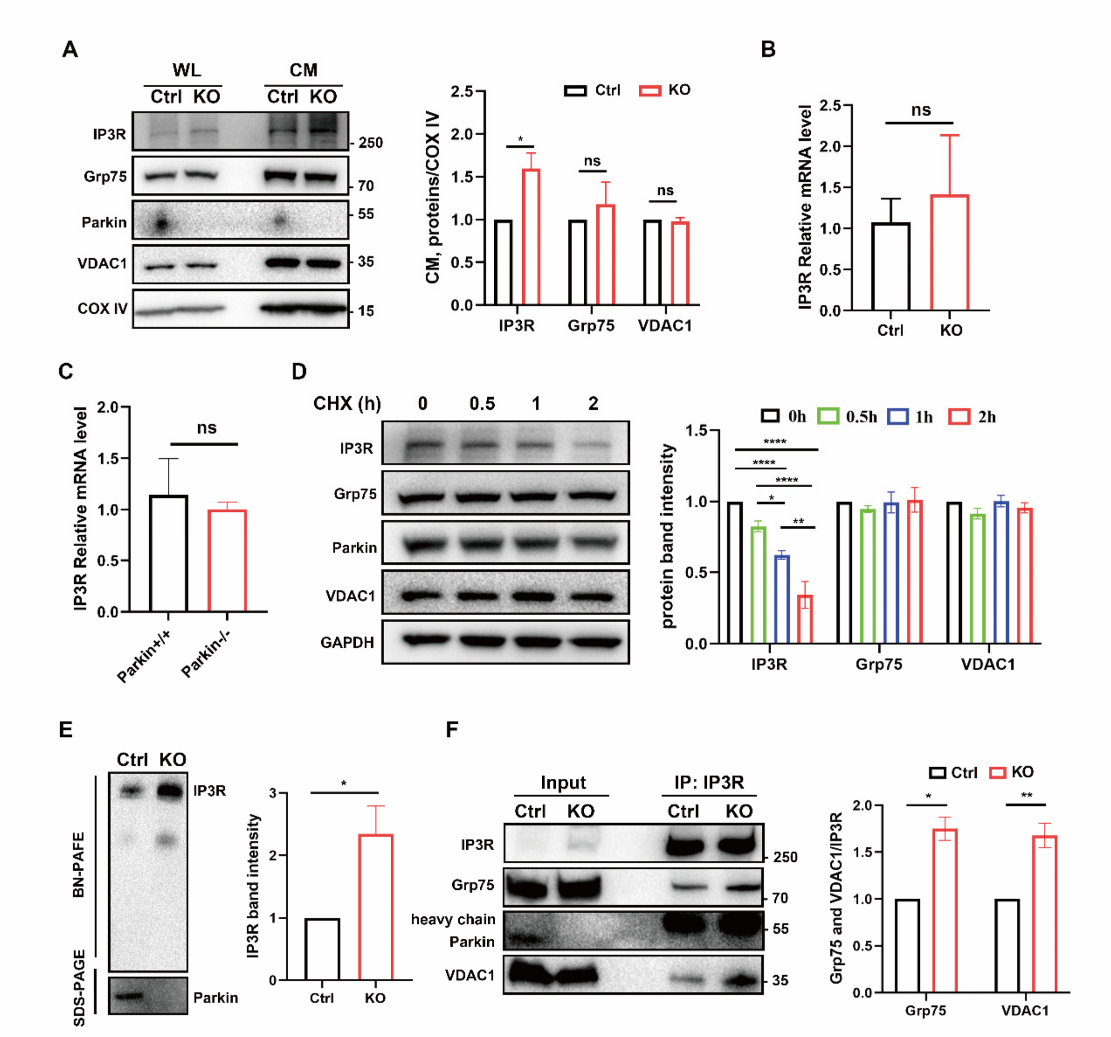

Figure S5 (A) Immunoblot analysis of chosen proteins in control and Parkin KO SH-SY5Y cells. Control and Parkin KO cells were fractionated into whole lysates (WL) and crude mitochondria (CM). Cytochrome c oxidase subunit IV (COX IV) was used as a loading control for CM proteins. Three independent experiments were conducted and were quantified. (B) Quantitative PCR (qPCR) analysis was performed to measure the expression of IP3R in control and Parkin KO M17 cells. (C) QPCR analysis was performed to measure the expression of IP3R in substantia nigra (SN) from WT and Parkin KO mouse brains. Three independent experiments were conducted. (D) Immunoblot analyses of time-dependent degradation of IP3R were detected after treatment with cycloheximide (CHX, 100 μg/ml, 0-2 hours) in Parkin overexpressing (OE) M17 cells. Three independent experiments were conducted and were quantified. (E) BN-PAGE analysis of the macrocomplex detected by IP3R in crude mitochondria from control and Parkin KO M17 cells. SDS-PAE immunoblot was used to analyze parkin in CM. Data are quantified from three independent experiments. (F) Immunoblot analysis of Grp75/VDAC1 was coimmunoprecipitated with IP3R antibody in control and Parkin KO SH-SY5Y cells. Three independent experiments were conducted and were quantified. Data were analyzed using two-tailed unpaired Student's t-test (B-C, E-F) and one-way analysis of variance (ANOVA) with Tukey's multiple comparisons test

(D). *P < 0.05; **P<0.01; ***P < 0.001.

A

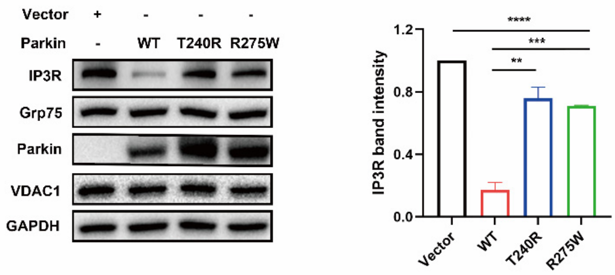

B

Parkin-mediated ubiquitinylation lysine residues in IP3R
K51, K52, K59, K70, K74, K77, K91, K100, K101, K109, K130, K136, K144, K168, K181, K225, K230, K235, K249, K258, K259, K279, K306, K350, K408,K412, K417, K424, K427, K451, K459, K462, K475, K501, K508, K517, K521, K569,K576,K583, K604, K608, K624, K643, K654, K669, K706, K711, K721, K725, K803, K827, K831, K858, K860, K862, K890, K909, K912, K917, K957, K963, K972, K974, K999, K1099, K1114, K1117, K1129, K1136, K1154, K1155, K1162, K1165, K1178, K1186, K1197, K1200, K1225, K1230, K1258, K1311, K1318, K1322, K1325, K1326, K1389, K1396, K1420, K1439, K1449, K1469, K1477, K1530, K1545, K1565, K1572, K1646, K1655, K1658, K1661, K1670, K1674, K1685, K1692, K1761, K1811, K1812, K1815, K1818, K1825, K1832, K1845, K1846, K1847, K1859, K1860, K1862, K1885, K1912, K1914, K1954, K2040, K2049, K2054, K2079, K2086, K2087, K2128, K2135, K2151, K2181, K2184, K2199, K2218, K2219, K2258, K2325, K2392, K2515, K2518, K2562, K2563, K2568, K2580, K2584, K2593, K2609, K2611, K2628, K2661, K2667, K2680, K2688, K2690

Figure S6 (A) Representative immunoblot and quantitative analyses of the degradation of IP3R from overexpressing vector, WT parkin, parkin T240R and R275W mutant M17 cells. Three independent experiments were conducted and were quantified. (B) Parkin-overexpressing (OE) M17 cells were transfected with an HA-Ub plasmid and treated with MG-132 for 6 h to enrich ubiquitinated proteins. Mass spectrometry analysis identified lysine residues on IP3R modified by Parkin-mediated ubiquitination. Data are expressed as means ± SEM based on three independent experiments. Data were analyzed using one-way analysis of variance (ANOVA) with Tukey’s multiple comparisons test (A). *P < 0.05; **P<0.01 ; ***P < 0.001.
